# The consequence of ATP synthase dimer angle on mitochondrial morphology studied by cryo-electron tomography

**DOI:** 10.1042/BCJ20230450

**Published:** 2024-01-29

**Authors:** Emma Buzzard, Mathew McLaren, Piotr Bragoszewski, Andrea Brancaccio, Holly C. Ford, Bertram Daum, Patricia Kuwabara, Ian Collinson, Vicki A.M. Gold

**Affiliations:** 1 Living Systems Institute, University of Exeter, Exeter, U.K.; 2 Faculty of Health and Life Sciences, University of Exeter, Exeter, U.K.; 3 Nencki Institute of Experimental Biology, Polish Academy of Sciences, Warsaw, Poland; 4 Institute of Chemical Sciences and Technologies ‘Giulio Natta’, National Research Council (CNR), Rome, Italy; 5 School of Biochemistry, University of Bristol, Bristol BS8 1TD, U.K.

**Keywords:** alphafold, ATP synthase, cryo-electron tomography, mitochondria, sub-tomogram averaging

## Abstract

Mitochondrial ATP synthases form rows of dimers, which induce membrane curvature to give cristae their characteristic lamellar or tubular morphology. The angle formed between the central stalks of ATP synthase dimers varies between species. Using cryo-electron tomography and sub-tomogram averaging, we determined the structure of the ATP synthase dimer from the nematode worm *Caenorhabditis elegans* and show that the dimer angle differs from previously determined structures. The consequences of this species-specific difference at the dimer interface were investigated by comparing *C. elegans* and *Saccharomyces cerevisiae* mitochondrial morphology. We reveal that *C. elegans* has a larger ATP synthase dimer angle with more lamellar (flatter) cristae when compared with yeast*.* The underlying cause of this difference was investigated by generating an atomic model of the *C. elegans* ATP synthase dimer by homology modelling. A comparison of our *C. elegans* model to an existing *S. cerevisiae* structure reveals the presence of extensions and rearrangements in *C. elegans* subunits associated with maintaining the dimer interface. We speculate that increasing dimer angles could provide an advantage for species that inhabit variable-oxygen environments by forming flatter, more energetically efficient cristae.

## Introduction

The F_1_F_o_ ATP synthase is a molecular motor ubiquitous to all living organisms, required for the essential conversion of an electrochemical gradient into the universal energy currency ATP [1]. The ATP synthase is composed of a catalytic F_1_ head connected to a membrane-embedded F_o_ motor by a central stalk; the entire assembly is visualised as a lollipop shape when examined by electron cryo-microscopy (cryoEM) [2,3]. The central stalk transmits the torque generated by the rotation of F_o_ to the F_1_ head, and a peripheral stalk acts as an elastic spring, ensuring malleable coupling between F_1_ and F_o_ [4]. Mitochondrial ATP synthases across species share the same complement of core subunits with varying nomenclature (Supplementary Table S1) [5,6]. In metazoans studied to date, the F_1_ head consists of α and β subunits, the central stalk of γ, δ and ε subunits, the peripheral stalk of *b*, *d*, F_6_ and oligomycin sensitivity conferral protein (OSCP) subunits, and the F_o_ motor contains the c-ring and subunit *a*.

Mitochondrial ATP synthases can assemble into dimers [7], of which there are four types [8]: Type I is present in both multicellular [9–11] and unicellular organisms [12] and type II–IV are present in various unicellular organisms [13–18], reviewed in [8]. When compared with type II–IV dimers, previously studied type I dimers contain an additional set of subunits at the dimer interface: *e*, *f*, *g*, *i*/*j*, *k* and 8 (Supplementary Table S1) [8]. Based on biochemical and imaging experiments, subunits *e* and *g* were shown to be essential for dimer formation [7,11,19–21]. Dimers of ATP synthases assemble into oligomeric rows (or ribbons) along the curved ridges of crista membranes, observed by cryo-electron tomography (cryoET) [9,11,14,22]. The formation of dimer rows is mediated by an ancestral motif in subunits *e* and *g* [20,21] with assistance from subunit *k* [5,23]. Dimer rows are required for crista membrane curvature, and thus maintenance of lamellar or tubular-shaped cristae [11,12,14,22,24]. Cristae undergo balloon-like deformation following the knockdown of subunits *e* or *g* [11] and on the disassociation of dimers into monomers [12], supporting the role of subunits *e* and *g* in influencing cristae morphology. Moreover, molecular simulations indicate that ATP synthase dimers have an innate propensity to induce membrane curvature [25]. This was confirmed experimentally when dimers reconstituted into liposomes spontaneously self-assembled into oligomeric rows to engender this curvature, maintaining identical dimer angles to those observed in whole mitochondria [22].

*In situ* structures of type I ATP synthase dimers have been determined from native membranes [10–12,22,26]. Mammals and fungi both display an average angle between the dimer heads of ∼86° [10]. Interestingly, higher-resolution single particle analysis of the purified bovine ATP synthase dimer reveals that dimer angles likely vary around this average (between 76° and 95°), depending on the catalytic state [27]. Further atomic-detail structures of purified mitochondrial type I ATP synthase dimers have also been determined from mammals (*Bos taurus* [23]) and fungi (*Saccharomyces cerevisiae* [28] and *Yarrowia lipolytica* [24]). Thus, whilst the structure and organisation of ATP synthase dimers have been studied across a range of different species, our knowledge of ATP synthases in invertebrates is lacking. The free-living nematode worm *Caenorhabditis elegans* is a well-established invertebrate model system for the study of cell and developmental biology [29], including the role of mitochondria in metabolism, health, disease and aging [30]. To complement *in vivo* physiological studies, intact mitochondria can be stably prepared [31,32] for biochemical and structural analyses [33]. Interestingly, studies have shown that nematodes lack the dimer-specific subunits *i*/*j*, *k* and 8 [34,35] found in mammals and fungi (Supplementary Table S1). Subunit 8 is encoded by one of two overlapping ATP synthase genes on the mitochondrial genome [36]. Proteins encoded on the mitochondrial genome are translated from essential genes [37,38]; thus it follows that subunit 8 is likely to be essential for respiration in mammals and fungi. The lack of dimer-specific subunits in *C. elegans* provides a unique opportunity to investigate how certain subunits influence ATP synthase dimer angles and mitochondrial morphology.

In this study, we employ cryoET and sub-tomogram averaging to determine the molecular architecture and organisation of the *C. elegans* ATP synthase in the membrane, revealing a novel average dimer angle of 105°. We also discover extra mass at the dimer interface compared with an equivalent *S. cerevisiae* structure [11]. We subsequently compare whole mitochondria from both *C. elegans* and *S. cerevisiae* to investigate the relationship between ATP synthase dimer angle and crista morphology. Finally, we use AlphaFold [39] and AlphaFold multimer [40] to predict how protein chains in the *C. elegans* ATP synthase dimer may be arranged. This allows us to analyse subunit differences at the dimer interface and postulate the cause of variations in angle. We speculate that an evolutionary divergence at the dimer interface and corresponding widening of the dimer angle may be an adaptation to more variable-oxygen environments.

## Results

### The molecular architecture of the *C. elegans* ATP synthase dimer

**Figure 1.**
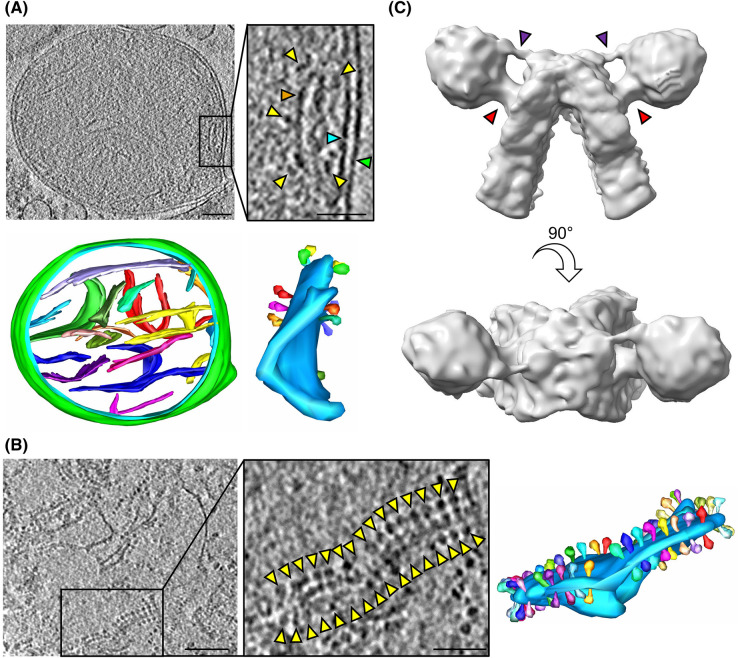
ATP synthase dimer rows, and sub-tomogram average of the ATP synthase dimer from *C. elegans*. (**A**) Tomographic slice through a whole *C. elegans* mitochondrion (top) and corresponding segmentation (bottom; outer membrane green, inner membrane light blue, multi-colour crista membranes). The boxed region shows an enlarged image of a single crista membrane, with green, blue and orange arrowheads indicating the outer, inner and crista membranes, respectively, and yellow arrowheads indicating ATP synthase F_1_ heads. The crista membrane is coloured light blue in the corresponding segmentation; each ATP synthase dimer pair is coloured differently. (**B**) Tomographic slice through *C. elegans* isolated crista membranes with boxed region showing enlarged image of a single crista membrane (left, arrowheads indicating ATP synthase F_1_ heads), and corresponding segmentation (right) coloured as in (**A**). Scale bars, 100 nm for tomograms, and 50 nm for enlarged views of crista membranes. (**C**) Sub-tomogram average of the *C. elegans* ATP synthase dimer. Upper panel shows side view with central and peripheral stalks indicated by red and purple arrows respectively, lower panel shows top-down view.

To determine the arrangement and molecular architecture of ATP synthase dimers in *C. elegans*, tomograms of whole mitochondria (Figure 1A) and of isolated crista membranes (Figure 1B) were collected and analysed. ATP synthases were unambiguously identified by the characteristic lollipop shape of the 10 nm diameter F_1_ heads positioned ∼10 nm away from the membrane. We confirmed the presence of oligomeric ATP synthase dimer ribbons, localised at the sharp curved ridges of crista membranes, in both samples (Figure 1A,B). Due to the obscuring presence of a dense matrix in whole mitochondria, many more dimers could be visualised in isolated crista membrane samples. Therefore, 3234 dimer pairs were extracted from the crista membrane data for sub-tomogram averaging. After classification, a map of the *C. elegans* ATP synthase dimer was determined from 1755 dimer pairs (Figure 1C, Supplementary Figures S1 and S2). Both the central and peripheral stalks were resolved clearly (Figure 1C).

**Figure 2.**
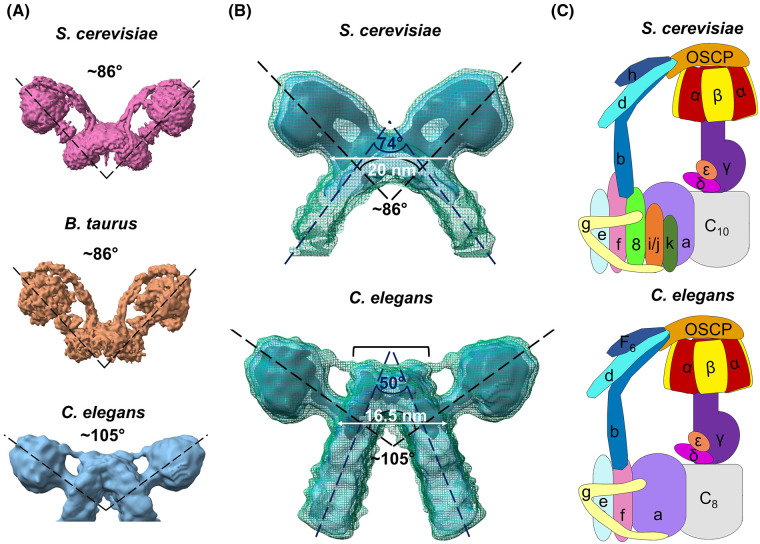
The *C. elegans* ATP synthase compared with other species. (**A**) Structures depicting the range of average dimer angles observed in *S. cerevisiae* (EMD-7067) [28], bovine heart (EMD-11436) [27], and *C. elegans* (this work (EMD-18991)), using the highest resolution structures available. (**B**) Direct comparison between *S. cerevisiae* (EMD-2161) [11] and *C. elegans* ATP synthase sub-tomogram averages, with the angle between F_1_ dimer heads, the angle of crista membrane curvature, and distance between the central stalks of each monomer indicated. A bracket highlights the extra mass at the *C. elegans* dimer interface not apparent in *S. cerevisiae*. Black, transparent blue and dark green mesh represent decreasing threshold levels for the averages. (**C**) Cartoon detailing the occurrence of ATP synthase subunits in *S. cerevisiae* and *C. elegans*, each labelled with corresponding nomenclature for the species (details in Supplementary Table S1).

Previous studies revealed a type I dimer angle of ∼86° across a range of mammalian and fungal species [10–12,22,26]. The architecture of the membrane-bound *C. elegans* ATP synthase dimer is unlike any other species studied so far, with an average angle of 105° between the dimer heads (Figure 2A). A comparison to the structure of the membrane-bound *S. cerevisiae* dimer [11] revealed that the wider dimer angle in *C. elegans* corresponds with a sharper angle of membrane curvature (50° compared with 74°) (Figure 2B). Accordingly, a shorter distance is measured between the two ATP synthase central stalks in *C. elegans* dimers compared with *S. cerevisiae* (16.5 nm compared with 20 nm), which has the effect of bringing the crista membranes closer together*.* Intriguingly, the dimer interface in the *C. elegans* map is also visually different from its *S. cerevisiae* counterpart (Figure 2B), and indeed all other type I dimers studied to date [10–12,24,26]. This difference is likely attributable to the different complement of dimer interface subunits present in *C. elegans* compared with *S. cerevisiae* (Supplementary Table S1, Figure 2C). We also analysed the inter-dimer distance and angle between dimer heads in consecutive dimers in the oligomeric rows. This revealed an inter-dimer distance of 12.5 nm and angle between dimer heads of 20° (Supplementary Figure S3). Despite differences in dimer angle, these values are consistent with those reported previously for the type II dimer from green algae (*Polytomella* sp.) [22], suggesting that dimer angle does not influence oligomerisation of ATP synthases into rows.

### A wider dimer angle in *C. elegans* corresponds to flatter, more lamellar cristae

**Figure 3.**
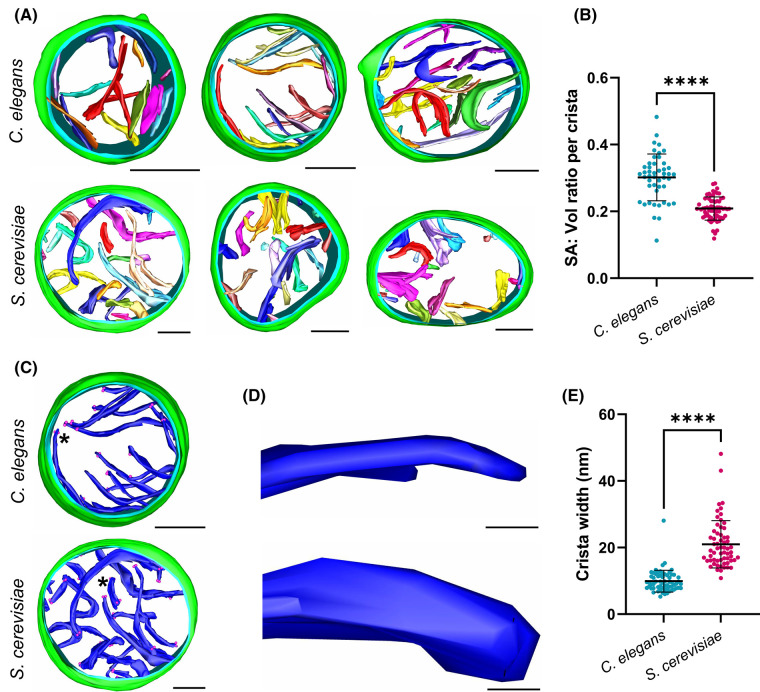
Morphology of mitochondria isolated from *C. elegans* and *S. cerevisiae*. (**A**) Tomographic segmentations of *C. elegans* and *S. cerevisiae* mitochondria are displayed (green, outer mitochondrial membrane; blue, inner mitochondrial membrane; multi-colour, crista membranes). See Supplementary Movie S1 (*C. elegans*) and Supplementary Movie S2 (*S. cerevisiae*). (**B**) The mean surface area to volume ratio per crista (*n* = 3 mitochondria for each organism, with *n* = 47 cristae for *C. elegans* and *n* = 63 cristae for *S. cerevisiae*) was calculated from the segmentations shown in (**A**). (**C**) A single tomographic segmentation from each organism is shown with all crista coloured blue. Pink dots indicate distances used to measure width. (**D**) Close up of a single crista membrane from each organism (location indicated by asterisks in (**D**)) to highlight the flatter crista morphology in *C. elegans* mitochondria compared with *S. cerevisiae*. (**E**) The mean crista width (*n* = 63 crista tips for *C. elegans* and *n* = 61 for *S. cerevisiae*) was calculated from the segmentations shown in (**A**). Error bars in (**B**) and (**E**) show the standard deviation of the mean and significance values were calculated using Welch's *t*-test for (**B**) or using the Mann–Whitney *U*-test for (**E**). ****P ≤ 0.0001. Scale bars in (**A**) and (**C**), 200 nm; in (**D**), 20 nm.

We hypothesised that the wider dimer angle associated with sharper membrane curvature in the *C. elegans* ATP synthase dimer (Figure 2B) would produce flatter cristae with a larger surface area to volume ratio. To test this, tomographic data of whole mitochondria from *C. elegans* and *S. cerevisiae* were collected and quantified. Qualitatively, *C. elegans* mitochondria appear to have more lamellar-shaped (or flatter) cristae, with sharp curved ridges, compared with mitochondria from *S. cerevisiae* (Figure 3A, Supplementary Movies S1 and S2). The surface area and volume of the crista membranes were quantified, to reveal that the surface area to volume ratio of the average crista membrane was significantly higher (∼1.5 fold, ****P ≤ 0.0001) in *C. elegans* than in *S. cerevisiae* (Figure 3B). In accordance with this, the average crista width in *C. elegans* was less than that observed in *S. cerevisiae* (Figure 3C–E), suggesting that dimer angle exerts influence on mitochondrial morphology at the level of membrane curvature.

Mitochondria are dynamic organelles, and crista morphology can be influenced by a wide range of factors such as metabolic state [41–44]. However, the average ATP synthase dimer angle remains consistent when imaged in membranes or on purification in detergent [10,27]. Our findings on crista width in whole mitochondria (Figure 3E) are corroborated by results obtained in isolated cristae containing either *C. elegans* or *S. cerevisiae* ATP synthase dimers (Figure 2B) [11]. This indicates that the dimer angle and corresponding angle of membrane curvature are consistent, irrespective of the method employed for sample preparation or analysis.

### A unique arrangement of subunits at the *C. elegans* dimer interface

We observed extra mass at the *C. elegans* dimer interface (Figure 2B) not previously observed in other type I structures determined to date [10,26]. Nematodes are missing subunit 8 [34] (Supplementary Table S1, Figure 2C), which plays a key structural role in other species [22,23,28,45] and is considered essential for respiration owing to its position on the mitochondrial genome [37,38]. Therefore, it is likely that other subunits undergo rearrangements at the dimer interface to compensate for the lack of subunit 8 in nematodes, which could contribute to the observed change of dimer angle. To explore this possibility, we performed multisequence alignments of dimer interface and peripheral stalk subunits with *C. elegans, S. cerevisiae* and *B. taurus* [46–48]*.* This revealed significant extensions in three *C. elegans* subunits located at the dimer interface (*e*, *f* and *g*), and in subunit *b* in the peripheral stalk (Supplementary Figure S4). A range of more subtle changes were also identified in subunits *d* and F_6_. To investigate if the changes in the dimer interface and peripheral stalk subunits could account for the extra mass observed at the dimer interface (Figure 2B), we built a homology model of the *C. elegans* ATP synthase. Prior to model building, mitochondrial targeting sequences were predicted and removed to allow structure prediction of mature protein subunits [49,50]. Mass spectrometry confirmed that the extensions identified by sequence alignment in the dimer interface subunits are present in the mature proteins and corroborate mitochondrial targeting sequence predictions (Supplementary Figure S5).

**Figure 4.**
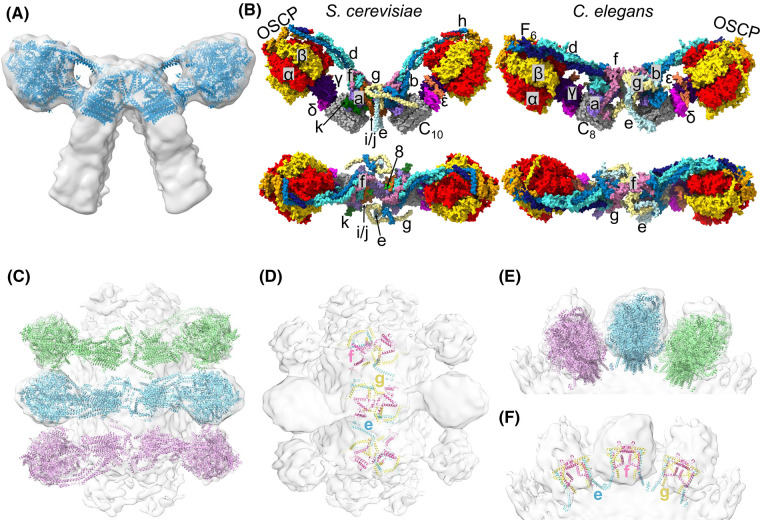
AlphaFold homology model of the *C. elegans* ATP synthase dimer. (**A**) Two ATP synthase monomers from the *C. elegans* homology model (helical representation) fitted into the sub-tomogram average of the *C. elegans* ATP synthase dimer. (**B**) Surface view of *S. cerevisiae* and *C. elegans* ATP synthase dimer models coloured by chain in side (top) and top-down (bottom) views. Subunits are annotated and shown as α, red; β, gold; γ, indigo; δ, magenta; ε, coral; *c*, grey; *a*, purple; *b*, blue; *d*, turquoise; F_6_, navy; OSCP, orange; *e*, pale blue; *f*, pink; *g*, yellow; *j*, brown; *k*, dark green; 8, lime. All subunits are labelled in the side views apart from subunit 8 which is buried. Only the dimer interface subunits are labelled in the top-down views. (**C**) Top-down view of the *C. elegans* ATP synthase dimer homology model fitted to the sub-tomogram average showing sequential dimer pairs (coloured differently) in a row. (**D**) As per (**C**), but exclusively showing dimer interface subunits *e*, *f* and *g*, labelled and coloured by chain as per (**B**). (**E**) and (**F**) show the same interactions as in (**C**) and (**D**), respectively, but viewed from the side of a dimer row.

The ATP synthase dimer is too large to predict the structure as a single multimer; therefore, we used AlphaFold [39] and AlphaFold multimer [40] to predict the structures of individual or small groups of subunits (Supplementary Figure S6 and Table S2). Considering that protein–protein interactions are likely important at the dimer interface, we predicted the dimer interface and peripheral stalk subunits both as individual subunits and as multimers. The peripheral stalk subunits were predicted successfully as a multimer, whereas the multimeric prediction for the dimer interface was poor. This could be explained by a limitation of the AlphaFold multimer, which does not take stepwise assembly of complexes into account, instead assembling all proteins into a multimeric complex simultaneously [40]. The result may also be attributable to the unique dimer interface in *C. elegans* compared with previously determined structures. The predicted *C. elegans* structures were then fitted sequentially into a scaffold provided by the *B. taurus* ATP synthase dimer (PDB 7AJB [27]) (Supplementary Figure S7 and Table S2). The atomic model of *B. taurus* was chosen as a scaffold, as like *C. elegans*, it is a metazoan, and contains an equivalent number of subunits (eight) in the c-ring [8]. The *C. elegans* ATP synthase dimer model was then split into monomers and each was fitted sequentially into our sub-tomogram average dimer map (Supplementary Figure S7, Figure 4A), improving the fit considerably (Supplementary Figure S8). The *C. elegans* homology model correlated well to the sub-tomogram averaging map (Supplementary Figures S8, S9 and Table S3), providing us with a useful working model to investigate potential protein–protein interactions and allow a comparison of *S. cerevisiae* and *C. elegans* ATP synthase dimers (Figure 4B).

To investigate the relative position of subunits required for the oligomerisation of dimers into rows, we fitted the *C. elegans* ATP synthase dimer model into a row of oligomeric dimer pairs along the curved edge of a crista membrane (Figure 4C–F). This reveals potential inter-dimer interactions mediated by subunit *e* (Figure 4D,F and Supplementary Figure S10). This is supported by recent work demonstrating the key role that subunit e plays in oligomerisation and row formation [21].

**Figure 5.**
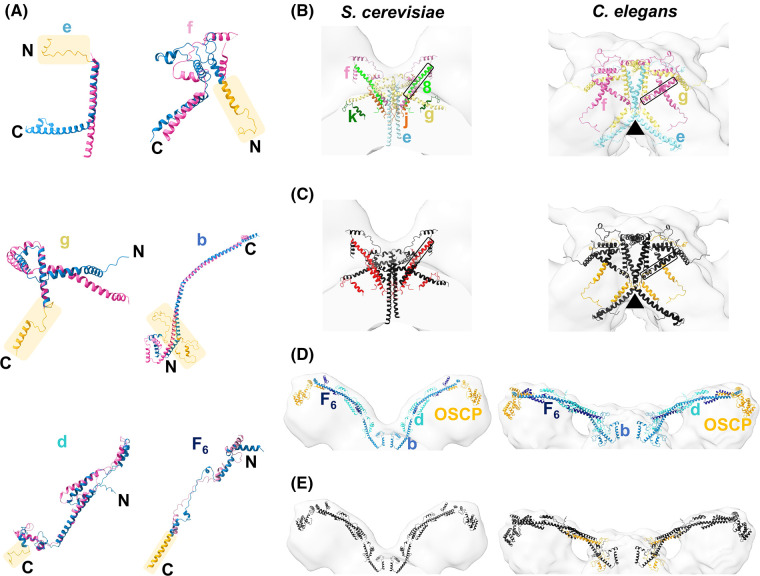
Comparison of the dimer interface and peripheral stalk in *C. elegans* vs *S. cerevisiae*. (**A**) Overlays of individual subunits at the dimer interface and peripheral stalk, where there are extensions in *C. elegans* subunits (AlphaFold predictions, blue) compared with *S. cerevisiae* (PDB 6B8H, pink) [28]. *C. elegans* subunit extensions are highlighted in orange. Since the *S. cerevisiae* atomic model for the ATP synthase dimer (PDB 6B8H) [28] does not contain complete density for subunit F_6_, the *S. cerevisiae* monomeric atomic model (PDB 6CP6) [70] was used to display a more complete *S. cerevisiae* chain for the overlay. (**B**) Left, dimer interface subunits in the *S. cerevisiae* atomic model (6B8H) [28] coloured by chain and fitted into an *S. cerevisiae* sub-tomogram average (EMD-2161) [11]. Right, dimer interface subunits in the *C. elegans* homology model coloured by chain fitted to the *C. elegans* sub-tomogram average. An alpha helix projecting from the dimer interface towards the peripheral stalk (subunit 8 in *S. cerevisiae* and subunit *f* in *C. elegans*) is boxed, and the sharp angle induced by subunits *e* and *g* in *C. elegans* indicated by a black arrowhead. (**C**) As per (**B**), but with all subunits coloured black, highlighting subunits missing in *C. elegans* relative to *S. cerevisiae* (*j*, *k* and 8) in red (left) and extensions in *C. elegans* subunits *e*, *f* and *g* relative to *S. cerevisiae* in orange (right). (**D**) Left, peripheral stalk subunits *b*, *d* and OSCP in the *S. cerevisiae* atomic model (PDB 6B8H) [28], and F_6_ from the monomeric atomic model (PDB 6CP6) [70], fitted to the *S. cerevisiae* sub-tomogram average (EMD-2161) [11]. Right, peripheral stalk subunits in the *C. elegans* homology model coloured by chain fitted to the *C. elegans* sub-tomogram average. (**E**) As per (**D**), but with all subunits coloured black, highlighting extensions in *C. elegans* subunits *b*, *d* and F_6_ relative to *S. cerevisiae* in orange. Subunits in (**B**–**E**) are annotated and shown as *b*, blue; *d*, turquoise; F_6_, navy; OSCP, orange; *e*, pale blue; *f*, pink; *g*, yellow; *j*, brown; *k*, dark green; 8, lime.

To analyse the dimer interface and peripheral stalk subunits in more detail, we overlayed the individual *C. elegans* predictions with their equivalents in the *S. cerevisiae* atomic model (PDB 6B8H) [28]. This revealed extensions in the *C. elegans* dimer interface subunits *e*, *f* and *g*, and peripheral stalk subunits *b*, *d* and F_6_ (Figure 5A), which is corroborated by our sequence alignments and mass spectrometry data (Supplementary Figures S4 and S5). The extra mass identified at the *C. elegans* dimer interface (Figure 2B) appears to be filled by a rearrangement of these extended subunits (Figure 5B–E). Specifically, extensions and rearrangements to subunits *e* and *g* in *C. elegans* relative to *S. cerevisiae* appear to induce a sharper membrane curvature (arrowhead in Figure 5B,C). The rearrangement of subunit *f* places its C-terminal alpha helix in the approximate position of the missing subunit 8 (boxed in Figure 5B,C). However, we cannot exclude the possibility that there are additional subunits yet unidentified in *C. elegans* that may also contribute to the dimer interface.

## Discussion

Owing to the essential and universal role of the ATP synthase across eukaryotic species, it is remarkable that the dimeric interface can be so variable [10,26]. Until now, the arrangement of ATP synthases in invertebrates was unknown, as was the correlation between dimer angle and whole mitochondrial morphology. In this work, a novel dimer angle for the ATP synthase from the nematode worm *C. elegans* was discovered. By comparing worm and yeast mitochondria, we correlated a wider ATP synthase dimer angle with flatter crista membranes. Since dimer row formation is known to be instrumental in the formation of curved ridges in crista membranes [11,12,14,22,24], it is consistent that dimer angle influences the extent of membrane curvature.

The *C. elegans* ATP synthase dimer shows clear extra mass at the dimer interface when compared with other determined structures, which can be attributed to changes in subunit composition. Using sequence analysis, we detected extensions in three *C. elegans* dimer interface subunits (*e*, *f* and *g*), an extension in the peripheral stalk component subunit *b*, and a range of more subtle changes in subunits *d* and F_6_. To investigate whether these could bulk out the width of the dimer interface, we built a homology model using AlphaFold [39] and AlphaFold multimer [40]. A recently proposed alternative method employs the prediction of subcomponent structures using AlphaFold multimer based on known assembly intermediates [51]. While conceptually advantageous for constructing a homology model of the ATP synthase dimer, accuracy decreases with increasing chain number and the approach has yet to be evaluated on protein complexes exceeding 30 chains [51].

The extensions in *C. elegans* ATP synthase subunits *e*, *f* and *g* appear to result in the rearrangement of proteins at the dimer interface relative to *S. cerevisiae*. In addition, the extension in peripheral stalk component subunit *b*, and the changes to subunits *d* and F_6_, appear to bulk out the width of the dimer interface. These alterations are associated with an increased level of membrane curvature in *C. elegans* compared with *S. cerevisiae*. In particular, the C-terminal extension in *C. elegans* subunit *g* is located in an area of sharp membrane curvature. Previous site-specific mutagenesis work of yeast subunit *g* revealed that the conserved C-terminal GXXXG motif is a key player in ATP synthase dimerisation and maintenance of lamellar crista membrane morphology [52]. Therefore, the *C. elegans* C-terminal extension to subunit *g* could be key in determining the extent of membrane curvature.

Some dimer interface subunits present in *S. cerevisiae* (*j*, *k* and 8) are absent from *C. elegans.* Whilst it cannot be completely excluded that yet unidentified subunits may substitute for one or more of these, we speculate that the absence of subunit 8 in worms [34] highlights an interesting evolutionary divergence. Subunit 8 is usually encoded by the mitochondrial genome, indicating that it is essential [37,38]. Additionally, subunit 8 appears to have a key structural role in joining the dimer interface to the peripheral stalk in all species studied to date [22,23,28,45]. We observed that an alpha helix at the C-terminus of *C. elegans* subunit *f*, projecting from the dimer interface to the peripheral stalk, appears to replace the missing density for subunit 8. It is, therefore, possible that subunit *f* has assumed the structural role of the missing subunit 8, supported by extensions in one or more of the dimer interface or peripheral stalk subunits close by (*b*, *d*, *e*, *f* or *g*).

The lack of subunit *k* in *C. elegans* (DAPIT in mammals), highlights another intriguing evolutionary deviation. In other species with type I dimers, subunit *k* is considered to play a key role in cross-linking dimers to generate oligomeric rows [5,23]. We speculate that the lack of subunit *k* in *C. elegans* may be compensated for by the extension in subunit *e*, which we observe to mediate tight inter-dimer interactions in dimer rows.

Mitochondria have evolved their highly convoluted crista membranes to increase their surface area [53], hence accommodating the maximum amount of respiratory chain complexes. This has made it possible for eukaryotic organisms to deal with higher energy demands than prokaryotes [53]. Whilst protein packing is dependent on the protein and lipid composition of the membrane [54], flatter cristae (in *C. elegans*) compared with wider cristae (in *S. cerevisiae*) could allow greater packing of respiratory chain complexes along the more lamellar membrane surfaces [10].

**Figure 6.**
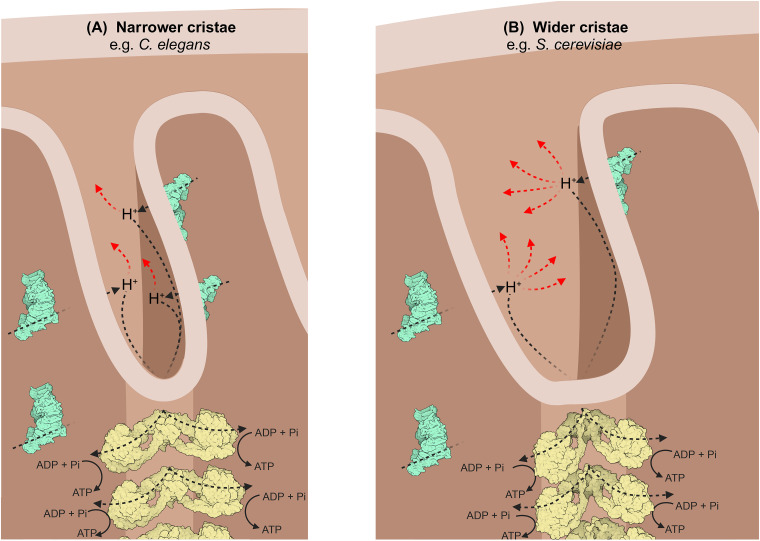
Schematic to illustrate the effect of crista shape on efficiency of proton diffusion from the proton pumping complexes (CI, III and IV) to ATP synthase. Dashed arrows indicate the direction of proton travel; black represents diffusion along membrane surfaces from ‘source’ to ‘sink’, red represents dissipation away from the membrane surface. Cartoons representing respirasomes and ATP synthase are derived from PDB depositions 5J4Z [73] and 6ZPO [23], respectively. The figure was created with Biorender.com. (**A**) Narrower cristae. In *C. elegans*, a wide angle between ATP synthase dimer heads (105°) induces a sharp angle of membrane curvature, producing lamellar cristae with the potential for increased protein packing and little opportunity for proton dissipation. (**B**) Wider cristae. In *S. cerevisiae*, and most other type I dimer species, a shallower angle is formed between the ATP synthase dimer heads (86°). This induces a less pronounced membrane curvature and therefore less lamellar-shaped cristae compared with *C. elegans*. There is a greater potential for protons to dissipate, and thus a greater distance to travel from source to sink.

It has been suggested that cristae serve as proton concentrators that facilitate a directed flow from the source (proton pumping complexes) to sink (ATP synthase) [9,10]; protons have been proposed to preferably migrate from source to sink along membrane surfaces. In mitochondria with narrower cristae, the higher membrane surface area to a given volume could lead to reduced dissipation of protons into the crista lumen [41], increasing the efficiency of ATP synthesis (Figure 6). This is supported by data establishing a correlation between narrower cristae and heightened metabolic activity in human cells [42–44].

We show that the ATP synthase dimer architecture defines the degree of cristae membrane curvature. We thus propose that the maximal rate of metabolism in an organism is dictated by its ATP synthase dimer angle. Narrow cristae in *C. elegans* could allow for both efficient packing of respiratory chain complexes, and reduced proton dissipation, allowing for maximum energy production in its soil-based habitat [55], where conditions range from hypoxic to near atmospheric [56,57]. In such a variable environment, narrower cristae may allow *C. elegans* to capitalise on oxygen when it is available. Interestingly, an even wider dimer angle (120°) has been identified in potato tubers [10], which like *C. elegans*, tend to reside in low-oxygen environments. Mammals, on the other hand, tend to have a more reliable source of oxygen and a wider dimer angle may not be a necessity. Energy must be invested in forming membrane curvature, and the more highly curved the membrane, the higher the cost [58]. In the case of mammals, the energetic cost of consistently maintaining a high degree of curvature may outweigh the benefit of increased respiratory efficiency.

In summary, we suggest that a range of ATP synthase dimer angles have evolved to meet the energetic needs of different organisms. Future studies geared towards investigating dimer subunit composition, angle and corresponding crista morphology across a range of species inhabiting different environments will be key in providing further support for this hypothesis. We demonstrate that the divergence in ATP synthase dimer architecture relative to yeast and mammalian systems makes *C. elegans* an ideal model system for further investigation of the role of dimer angle in mitochondrial physiology, health and disease.

## Materials and methods

All standard reagents were purchased from Sigma–Aldrich (Burlington, U.S.A.).

### *C. elegans* and *S. cerevisiae* culture

The *C. elegans* N2 Bristol strain was maintained at 20°C on 60 mm nematode growth medium (NGM) plates seeded with *Escherichia coli* OP50. For large-scale preparations, a semi-synchronised population of *C. elegans* (achieved by starving so that they entered the dauer stage) [59,60] were grown in a liquid suspension of *E. coli* NA22 in S-basal complete medium [61] at 20°C, shaking at 200 rpm for 3 days to achieve adults. For further details see [32]. *S. cerevisiae* ‘Bakers's yeast’ S288C derivative strains YPH499 were cultured at 19–24°C in YPGal or YPG medium (1% w/v yeast extract, 2% w/v bactopeptone, 2% w/v galactose or 3% w/v glycerol) until OD 2–2.5 was reached. For further details see [62].

### Mitochondrial isolation

*C. elegans* and *S. cerevisiae* were both harvested from liquid cultures by low-speed centrifugation. *C. elegans* preparation required an additional sucrose flotation step to remove debris. To soften the *C. elegans* cuticle, the pellets underwent collagenase treatment (1 U/ml collagenase, 100 mM Tris–HCl pH 7.4 and 1 mM CaCl_2_), whilst *S. cerevisiae* pellets underwent dithiothreitol (10 mM DTT, 100 mM Tris-SO_4_ pH 9.4) and zymolyase treatment (4.5 mg/g zymolyase, 1.2 M sorbitol, 20 mM potassium phosphate, pH 7.4) to disrupt the cell wall. Pellets from both species were re-suspended in homogenisation buffers. For *C. elegans*, this was STEG/M (220 mM mannitol, 70 mM sucrose, 5 mM Tris–HCl pH 7.4 and 1 mM EGTA) supplemented with 1 mM PMSF in methanol and 1% (w/v) fatty acid-free BSA. For *S. cerevisiae* the homogenisation buffer contained 0.6 M sorbitol, 10 mM Tris–HCl pH 7.4, 1 mM PMSF, 0.2% (w/v) BSA, 2 mM magnesium acetate. The re-suspended *C. elegans* or *S. cerevisiae* samples were homogenised in a glass-Teflon Potter homogeniser to break open cells. Both samples were subsequently spun at low speed (750–3000×***g*** for 5–15 min) to remove cell debris and nuclei, and then at higher speed (12 000×***g*** for 15 min) to pellet mitochondria. Purified mitochondria were re-suspended in buffers that were optimised to maintain intact mitochondria: STEG/M for *C. elegans* or 250 mM sucrose, 2 mM magnesium acetate, 10 mM MOPS-KOH pH 7.2 for *S. cerevisiae*.

### Mitochondrial crista membrane isolation

Crista membranes used for the sub-tomogram averaging experiments were generated by successive freeze–thaw cycles of mitochondria at −80°C. To purify mitochondrial membranes from other cellular material, membrane extracts were incubated for 1 h at 4°C with an anti-NDUFS3 primary antibody (ab14711; Abcam) against the matrix arm of complex I from *C. elegans*, followed by a 3 h incubation with an anti-mouse secondary conjugated to a quantum dot emitting at 625 nm (Q22085; Invitrogen). Crista membranes were separated from unbound antibodies and other cellular material on an Optiprep gradient with 10 layers (200 µl volume each) ranging from 0 to 27% v/v of iodixanol in STEG/M buffer, by centrifugation at 80 000×***g*** for 30 min at 4°C using a TLS-55 rotor (Beckman Coulter Inc., Miami, FL, U.S.A.)*.* Crista membranes were identified and removed based on fluorescence under a UV lamp. Samples were then diluted in STEG/M buffer to wash out the iodixanol, and spun at 20 000×***g*** for 15 min at 4°C to pellet the membranes. The enriched cristae were again re-suspended in the STEG/M buffer.

### Electron cryo-tomography

Whole mitochondria or crista membranes were mixed 1:1 with 10 nm gold fiducials (Aurion, Wageningen, The Netherlands), applied to glow-discharged holey carbon EM grids (Quantifoil, Jena, Germany), and blotted for 5–6 s, followed by plunge-freezing in liquid ethane using a Vitrobot Mark IV (ThermoFisher, MA, U.S.A.) for *C. elegans*, or a home-made device for whole *S. cerevisiae* mitochondria. Pre-screening of *C. elegans* grids was carried out using an FEI Tecnai Spirit 120 kV microscope (ThermoFisher), with a OneView CCD Camera (Gatan, Pleasanton, U.S.A.). CryoET was performed using the same microscope for whole mitochondria, or using a 200 kV Talos Arctica (ThermoFisher) for crista membranes, equipped with a K2 direct electron detector camera and a GIF Quantum LS energy filter (Gatan). CryoET of whole *S. cerevisiae* mitochondria was performed using a 300 kV Titan Krios (ThermoFisher), K2 direct electron detector camera and a GIF Quantum LS energy filter (Gatan). Single tilt image series’ (±60, step size 1.5°–2°) were collected at −5 to −8 µm underfocus at nominal magnification of 21 000× for whole mitochondria and 39 000× for crista membranes, corresponding to 5.4 and 3.58 Å pixel sizes, respectively, for *C. elegans*, or 26 000× for whole mitochondria from *S. cerevisiae*, corresponding to a 4.51 Å pixel size*.* The total dose per tomogram was ∼120 e^−^/Å^2^ for whole mitochondria, and ∼80 e^−^/Å^2^ for isolated cristae. Tomograms were aligned using the gold fiducials in IMOD (University of Colorado, U.S.A.) [63] and volumes reconstructed via weighted back-projection. Contrast was enhanced by nonlinear anisotropic diffusion (NAD) filtering [64], followed by manual segmentation, also in IMOD. ImageJ [65] was used to generate movies of segmentations generated in IMOD.

### Sub-tomogram averaging

3234 *C. elegans* ATP synthase dimers were picked manually in IMOD, using NAD-filtered tomograms. Sub-volumes containing the ATP synthase dimer were then extracted from tomograms that had not been NAD filtered. These sub-volumes were CTF corrected and imported into RELION 3.1 [66] using the approach and script described in [67]. A reference-free initial model was generated using 3× binned sub-volumes and 2481 dimers were selected by 2D classification for an unbinned refinement. Finally, 1755 dimers were selected from a 3D classification of this refined model to enter a final round of refinement and post-processing, resulting in a 36 Å resolution map. Supplementary Figure S1 details the full workflow.

### Homology model generation

AlphaFold was used to predict five structural models of each ATP synthase subunit in *C. elegans* based on their mature protein sequence [39]. Mature sequences were determined using MitoFates [49] and TargetP-2.0 [50] to predict mitochondrial targeting sequences. Predicted cleavage sites were interrogated manually to confirm whether consensus site rules were adhered to. If the mean probability of cleavage in both softwares was >0.5, and a valid consensus sequence identified, the corresponding mitochondrial targeting sequence was removed before submitting the FASTA file to AlphaFold for structure prediction. All ATP synthase subunits known to be present in *C. elegans* were included, excepting a putative homologue of subunit *j*, on account of its poor alignment with other homologues, and absence of any corresponding peptides in mass spectrometry analysis of the *C. elegans* dimer. The structures of peripheral stalk subunits *b*, *d* and F_6_ were predicted using AlphaFold multimer [40]. The models for each subunit with the highest average pLDDT score were fitted sequentially to a scaffold provided by the atomic model of the *B. taurus* ATP synthase dimer (PDB 7AJB) [27] in ChimeraX [68] using the Matchmaker tool. *B. taurus* was chosen as an organism to provide the scaffold because, like *C. elegans*, it is also a metazoan and contains the same number of subunits (eight) in the C-ring [8]. Both monomeric and dimeric *B. taurus* ATP synthase structures exist in different catalytic states [23,27]; there were no significant differences apparent in the homology model depending on the model chosen. Therefore, we selected the *B. taurus* dimer as a scaffold [27], enabling clearer visualisation of differences in dimer angles between species. Where a subunit had more than one isoform, the version with the highest pLDDT score was used. In the case of subunit *b*, the isoform with the highest pLDDT score is also the only isoform expressed in somatic tissues [69]. Due to the differences in dimer angle between the *C. elegans* and *B. taurus* ATP synthases, the resulting dimeric structure was divided into monomers to fit the *C. elegans* homology model into the *C. elegans* map accurately. This was performed sequentially using the ‘fit in volume’ tool in ChimeraX [68]; the workflow is shown in Supplementary Figure S7 and the result in Supplementary Figure S8. To check the reliability of the fit, the resulting homology model was converted into an MRC map using the molmap command in ChimeraX [68]. This map could then be fitted into the sub-tomogram average map of the *C. elegans* dimer for comparison in Supplementary Figure S9. The yeast monomeric atomic model (PDB 6CP6) [70] was used for analysis in Figure 5A since it contains a more complete chain for F_6_ than the dimeric atomic model from yeast (PDB 6B8H) [28].

### Mass spectrometry

The ATP synthase was purified from *C. elegans* mitochondria using a method described previously [71,72], and analysed by Nano-LC mass spectrometry. Briefly, isolated mitochondria were solubilised and mixed with a His-tagged inhibitor protein IF_1_. This suspension was applied to a Nickel column to capture inhibited ATP synthase. The fraction most enriched in ATP synthase subunits was taken for mass spectrometry analysis. Further details are given in Supplementary Material.

## Data Availability

The sub-tomogram averaging maps generated in this study have been deposited in the Electron Microscopy Data Bank (EMDB) under accession code EMD-18991. The raw data have been deposited to the Electron Microscopy Public Image Archive (EMPIAR) under accession number EMPIAR-11542. The Source Data accompanying Figure 3B,E can be found in the accompanying Source Data file.

## Abbreviations

cryoET: cryo-electron tomography
NAD: nonlinear anisotropic diffusion
OSCP: oligomycin sensitivity conferral protein
