# Supplementary material for: The consequence of ATP synthase dimer angle on mitochondrial morphology studied by cryo-electron tomography
Source: Biochem J. 2024 Jan 29;481(3):161–75. doi: 10.1042/BCJ20230450 (PMC10903453; doi:10.1042/BCJ20230450)
Supplement: Supplementary Material 1 [file BCJ-481-161-s1.pdf]

Supplementary Material

The consequence of ATP synthase dimer angle on mitochondrial morphology studied by cryo-electron tomography

Emma Buzzard, Mathew McLaren, Piotr Bragoszewski, Andrea Brancaccio, Holly Ford, Bertram Daum, Patricia Kuwabara, Ian Collinson & Vicki A.M. Gold.

This PDF file includes:

Supporting text

Figures S1 to S10

Tables S1 to S3

Legends for Supplementary Movies 1 and 2

SI References

Other supporting materials for this manuscript include the following:

Supplementary Movies 1 and 2

ATP synthase purification from *C. elegans* mitochondria

Purification of IF_1

C. elegans ATP synthase was purified using His-tagged IF_1 as bait, following a scaled-down protocol designed for purification of bovine dimers (1,2). Residues 1-60 of the *C. elegans* F-ATPase inhibitor protein IF_1 fused to a hexa-histindine tag (ceI1-60His), were overexpressed from a pRSFDuet plasmid in *E. coli* BL21 (DE3) and purified by affinity chromatography on a 5 mL Nickel-Sepharose column (Cytiva) attached to an ÄKTA purification system (Cytiva). Fractions enriched in IF_1 were concentrated to ~50 mg/mL with a VivaSpin concentrator (molecular weight cut-off 3 kDa; Sartorius).

Purification of ATP synthase

C. elegans mitochondria were washed in a phosphate buffer (50 mM sodium hydrogen phosphate, 100 mM sucrose and 0.5 mM EDTA) and then centrifuged at 13,700 *x g* for 45 minutes at 4°C. This wash step was repeated twice to remove endogenous *C. elegans* IF_1. Phosphate-washed mitochondria (~16 mg) were solubilised for 30 minutes at 18°C at 7.65 mg/ml with digitonin (0.92% w/v) and DDM (0.76% w/v). The resulting extract was centrifuged at 24,000 *x g* for 20 minutes at 4°C, and ceI1-60His was added to the supernatant at 2.7 µg per 1 mg mitochondria to form ATPase:ceI1-60His complexes. A solution of 200 mM ATP, 200 mM $MgSO_4$, and 400 mM Trizma (pH 8.0) was also added at 15 µl/ml before incubating for 15 minutes at 37°C, with further additions of this solution being added at 5 minute intervals. Precipitate was removed by centrifugation at 24,000 *x g* for 10 minutes at 4°C. NaCl and imidazole were added to the clarified sample to reach final concentrations of 150 mM and 25 mM respectively. This final extract was applied to a 1 mL HisTrap FF Nickel Column (Cytiva) installed on an ÄKTA purification system (Cytiva) and equilibrated in a buffer containing 20 mM Tris, pH7.4, 150 mM NaCl, 2 mM ATP, 2 mM $MgSO_4$, 10% (v/v) glycerol, 0.1% (w/v) glyco-diosgenin (GDN) and a 0.1 mg/mL phospholipid mix. The ATPase:ceI1-60His complexes were eluted from the column by addition of a linear gradient of imidazole up to 500 mM over 10 mL. 0.5mL fractions were collected and run on an SDS-PAGE gel to confirm which fractions contained the ATPase:ceI1-60His.

Nano-LC Mass Spectrometry

The sample of ATP synthase was run on a 10% SDS-PAGE gel until the dye front had migrated approximately 1 cm into the separating gel. The gel lane was then excised as a single slice and subjected to in-gel tryptic digestion using a DigestPro automated digestion unit (Intavis Ltd.). The resulting peptides were fractionated using an Ultimate 3000 nano-LC system in line with an Orbitrap Fusion Lumos mass spectrometer (Thermo Scientific). Spectra were acquired with Xcalibur 3.0 software (Thermo Scientific).

The raw data files were processed and quantified using Proteome Discoverer software v2.1 (Thermo Scientific) and searched against the UniProt *C. elegans* database (downloaded October 2022; 26728 sequences) using the SEQUEST HT algorithm. Search criteria included oxidation of methionine (+15.995Da), acetylation of the protein N-terminus (+42.011Da) and methionine loss plus acetylation of the protein N-terminus (-89.03Da) as variable modifications and carbamidomethylation of cysteine (+57.021Da) as a fixed modification. Searches were performed with full tryptic digestion and a maximum of 2 missed cleavages were allowed. The reverse database search option was enabled and all data was filtered to satisfy false discovery rate (FDR) of 5%.

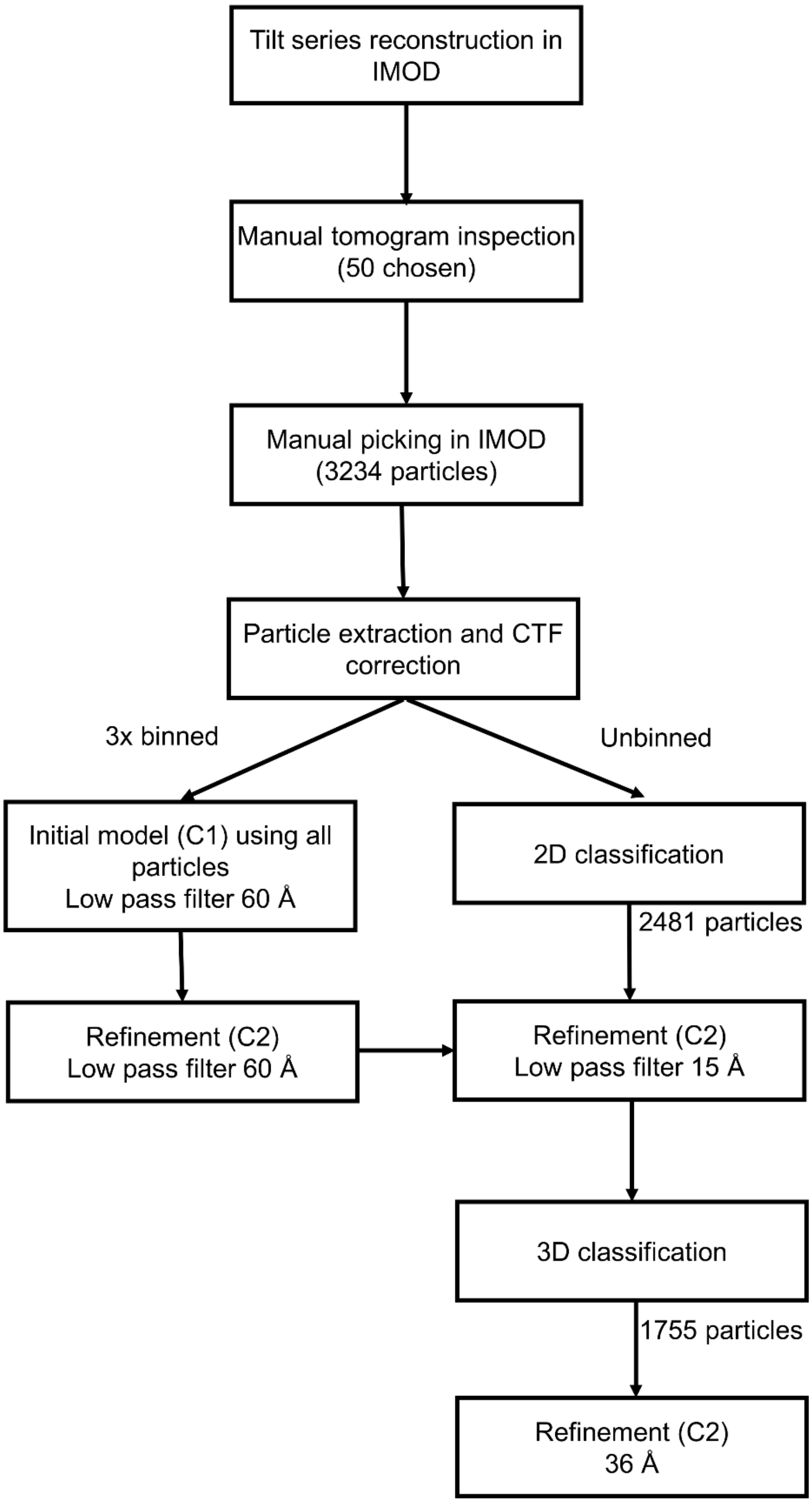

Figure S1. Flow chart of tomogram processing and sub-tomogram averaging using IMOD and RELION.

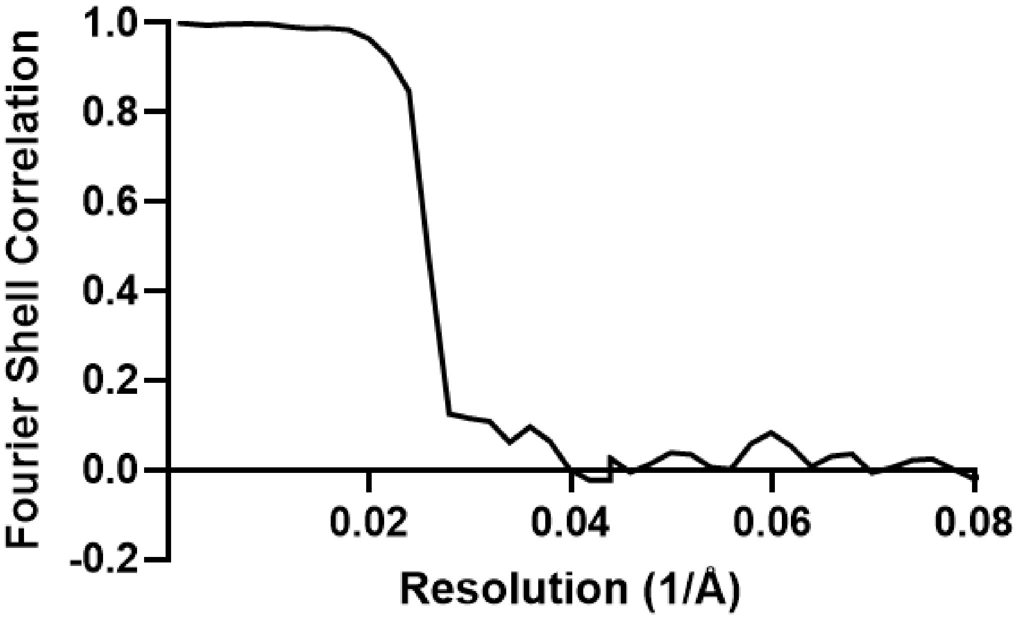

Figure S2. Fourier Shell Correlation (FSC) for the *C. elegans* ATP synthase sub-tomogram averaging map. The corrected FSC curve is an output from RELION 3.1 with a reported resolution of 36 Å according to the 0.143 criterion.

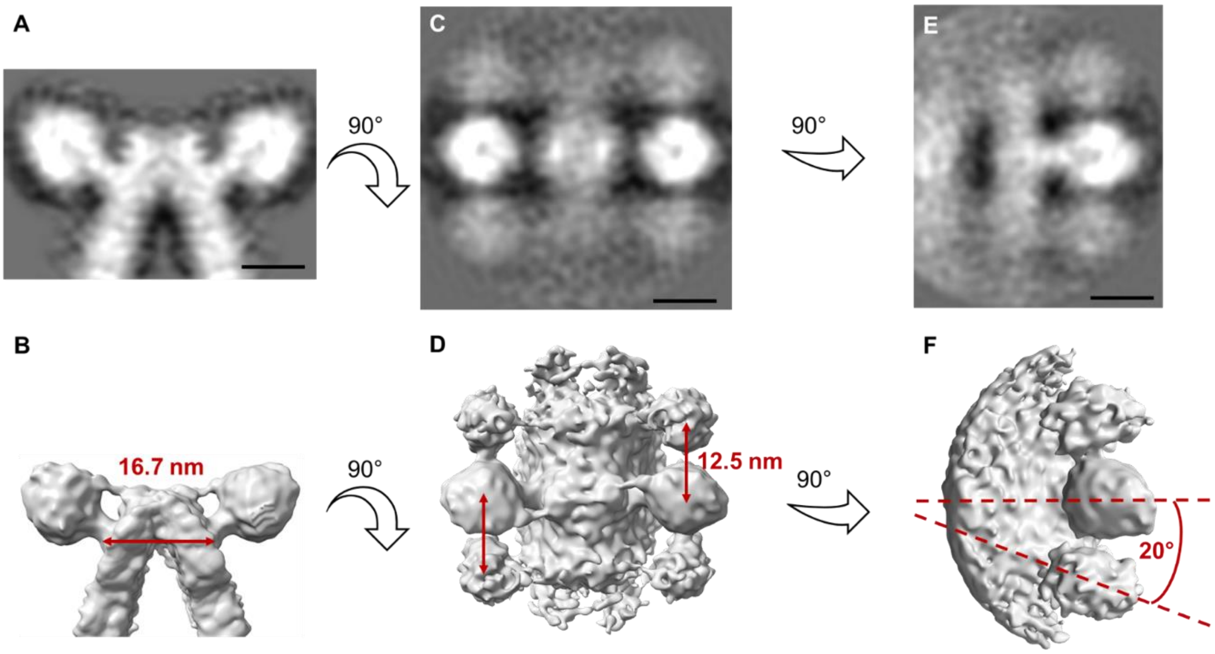

Figure S3. Inter-dimer distance and angle between consecutive dimer heads in oligomeric rows of *C. elegans* ATP synthase dimers. (A) 2D projection showing a side view of a masked map of the *C. elegans* ATP synthase dimer. **(B)** Side view shown in 3D, with distance between central stalks indicated. **(C)** 2D projection showing top-down view of an unmasked map of the *C. elegans* ATP synthase dimer. **(D)** Top-down view in 3D with inter-dimer distance indicated. **(E)** 2D projection showing side view (rotated 90° compared to A) of an unmasked map of the *C. elegans* ATP synthase dimer. **(F)** Side view in 3D, with inter-dimer angle indicated. All indicated measurements were made in IMOD. Scale bars, 10 nm.

Subunit e

```
tr|Q21732|Q21732_CAEEL        MSAPLKHPNAVVLQPPTVTISPLIRFGRYAALSLGVVYGFFRLRQIREYHADIREWDH-E    59
sp|P81449|ATPJ_YEAST          --------------------MSTVNVLRYSALGLGLFFGFRNDMILKCNAKKKEEQAQYE    40
sp|Q00361|ATP5I_BOVIN         -------------MVPPVQVSPLIKLGRYSALFLGMAYGAKRYNYLKPRAEEERRLAA-E    46
                                                     :.. **:** **: :*  .   ::    . ..    *

tr|Q21732|Q21732_CAEEL        KAVAAAEEAAKKKKWLAKD-----------EMRYLMQVVNIPFEEGVKQFGVADLYKED-    107
sp|P81449|ATPJ_YEAST          EKLKLVEEAK---KEYAKLHPVVTPKDVPANASFNLEDPNIDFERVIL--NAVESLKEAS    95
sp|Q00361|ATP5I_BOVIN         EKKKRDEQK-RIERELAEA-----------QEDTILK-----------------------    71
                              :     *:     :  *:            :    ::

tr|Q21732|Q21732_CAEEL        -    107
sp|P81449|ATPJ_YEAST          T    96
sp|Q00361|ATP5I_BOVIN         -    71
```

Subunit f

```
sp|Q22021|ATPK_CAEEL          MAWFRPPPPHTQLRPWVPDAIFIPISRAVERVGVFFYNRVLNKTEVGLFDKRWNKNVHGP  60
sp|Q06405|ATPK_YEAST          MI-------------------F---KRAV---STLIPPKVVSS-----------------  18
sp|Q28851|ATPK_BOVIN          ------------------------------------------------------------  0

sp|Q22021|ATPK_CAEEL          YCHWRYYGKLDTKFMDVKLGDLPAWM------ARREKTPSAFYNE--FMRNIWRVHNLYY  112
sp|Q06405|ATPK_YEAST          ----------------KNIGSAPNAKRIANVVHFYKSLPQGPAPAIKANTRLARYKAKYF  62
sp|Q28851|ATPK_BOVIN          --MASVVPLKEKKLLEVKLGELPSWI------LMRDFTPSGIAGA--FQRGYYRYYNKYV  50
                                               ::*. *            .  *..            *    *

sp|Q22021|ATPK_CAEEL          SGPVYNNTVKVIFRF-----IFAYSFLNWLVKSHRYVDFQKTMYHW        153
sp|Q06405|ATPK_YEAST          DGDNA--SGKPLWHFALGIIAFGYSMEYYFHLR----HHKGAEEH-        101
sp|Q28851|ATPK_BOVIN          NVK--KGSIAGLSMV-----LAAYVFLNYCRSYKELKHERLRKYH-        88
                              .      :   :  .       .* :  :        . :    *
```

Subunit g (isoform 2)

```
sp|Q18803|ATPL2_CAEEL         MAAPKLGFFEKIANLTGALYRHQHAQFPRR-------F-AILKAVGKHELAPPRQADWPA 52
sp|Q12233|ATPN_YEAST          -------MLSRIQNYTSGLVSKANLLSSKALYYGKVGAEISKQIYLKEGLQPPTVAQFKS 53
sp|Q28852|ATP5L_BOVIN         ----MAEFVRNLAEKAPALVNAAVTYSKPR-------L-ATFWYYAKVELVPPTPAEIPT 48
                                     :. .: : : .*                           *  * **  *:  :

sp|Q18803|ATPL2_CAEEL         IKADWAKV-QSF-----IQTGGYKNLSIREGLVYTAVTLEVVFWFFVGEMIGRRYIFGYL 106
sp|Q12233|ATPN_YEAST          VYSNLYKQSLNFALKPTEVLSCLKNIQKNELLKYGAYGIQLIGFYSVGEIIGRRKLVGYK 113
sp|Q28852|ATP5L_BOVIN         AIQSLKKI-INS-----AKTGSFKQLTVKEALLNGLVATEVWMWFYVGEIIGKRGIIGYD 102
                                 .  *   .         .  *::  .* *       ::  :: ***:**:* :.**

sp|Q18803|ATPL2_CAEEL         VPADYVSKSTKKTVKEQEALAALEN      131
sp|Q12233|ATPN_YEAST          HH-----------------------      115
sp|Q28852|ATP5L_BOVIN         V------------------------      103
```

Subunit b (isoform 2)

```
sp|Q19126|AT5F2_CAEEL   MSLSRCLPLGQNARVIIIPARLAHAASTQAAAATDDAPNFFQKLAHRFQGVPLKGEAHAP 60
sp|P05626|ATPF_YEAST    MSM------------------SM-----------------------GVRGLALR---SVS 16
sp|P13619|AT5F1_BOVIN   MLS------------------RV-----------------------VLS--AAA---AAA 14
                        *                    :                         .          .

sp|Q19126|AT5F2_CAEEL   KSMFEDCNKEWSAPEPLPAIPKDFKEHPDRDLVNYPYPARPMYPPKSRLLMMPDSWFTPF 120
sp|P05626|ATPF_YEAST    KTLFSQG---VRCPSMVIGA----RYM-SSTPEKQT---DPKAKANSIINAIPGN---NI 62
sp|P13619|AT5F1_BOVIN   PSLKNAA---LLGPGVLQAT----RIFHTGQPSLAPVPPLPEHGGKVRFGLIPEEFFQFL 67
                         :: .        *  : .     :               *    :  :  :* .    :

sp|Q19126|AT5F2_CAEEL   QKVTGVSGPYLFFGGLFAFLVNKELWVFEEQGHMTVGWILFYLLVTRTAGYKIDQGLYNG 180
sp|P05626|ATPF_YEAST    LTKTGVLG---TSAAAVIYAISNELYVINDESILLLTFLGFTGLVAKYLAPAYK-----D 114
sp|P13619|AT5F1_BOVIN   YPKTGVTGPYVLGTGLILYLLSKEIYVITPETFSAISTIGFLVYIVKKYGASVG-----E 122
                           *** *      . . : :.:*::*:  :    :  : *   :.:  .

sp|Q19126|AT5F2_CAEEL   YQERVNF--------FKGLIQEDLKEAVEFKKTSAKQTESLNSIKESYPTALKESMALQL 232
sp|P05626|ATPF_YEAST    FADARMKKVSDVLNASRNKHVEAVKDRID----SVSQLQNVAETTKVLFDVSKETVELES 170
sp|P13619|AT5F1_BOVIN   FADKLNEQKIAQLEEVKQASIKQIQDAID----MEKSQQALVQKRHYLFDVQRNNIAMAL 178
                        : :             :    : ::: ::      .. : : .  .    . ::.: :

sp|Q19126|AT5F2_CAEEL   EATYRKNVQSVATELKRRIDYLKETEESKARVEREQLLKLINSEVDKEFSDRSFKDKYLQ 292
sp|P05626|ATPF_YEAST    EAFELKQKVELAHEAKAVLDSWVRYEASLRQLEQRQLAKSVISRVQSELGNPKFQEKVLQ 230
sp|P13619|AT5F1_BOVIN   EVTYRERLHRVYREVKNRLDYHISVQNMMRQKEQEHMINWVEKRVVQSISAQQE-KETIA 237
                        *.   :.   :  * *  :*     :    : *:.:: : : ..* ..:.  .  .: :

sp|Q19126|AT5F2_CAEEL   NAIQQLKGLNVQL------  305
sp|P05626|ATPF_YEAST    QSISEIEQLLSKLK-----  244
sp|P13619|AT5F1_BOVIN   KCIADLKLLSKKAQAQPVM  256
                        :.* ::: *  :
```

Subunit d

```
tr|Q17763|Q17763_CAEEL   MSGAAKRVATSSVNWSKLAER--LVPEHAAELTRVKGVSGTFQSAVSQLPADLPKIDFAA   58
sp|P30902|ATP7_YEAST     --MSLAKSAANKLDWAKVISSLRITGSTATQLSSFKKRNDEARRQLLELQSQPTEVDFSH   58
sp|P13620|ATP5H_BOVIN    --MAGRKLALKTIDWVAFGEI--IPRNQKAVANSLKSWNETLTSRLATLPEKPPAIDWAY   56
                            :  : * ..::*  . .   :  .  :  .. *  .      :  *  .   :*::

tr|Q17763|Q17763_CAEEL   LKKALPAHSA--VLDSLQKQYESVKIPYGEVPAEYL---------KEVDQWVDYNNARIK   107
sp|P30902|ATP7_YEAST     YRSVLKNTSVIDKIESYVKQYKPVKIDASKQLQ-------------VIESFEKHAMTNAK   105
sp|P13620|ATP5H_BOVIN    YKANV-AKAG--LVDDFEKKFNALKVPIPEDKYTAQVDAEEKEDVKSCAEFLTQSKTRI-   112
                          :  :   :    ::.  *::: :*:   :                   .:     :.

tr|Q17763|Q17763_CAEEL   LHEVKVADGLQEAKKVEEKWAKAPPVEHFDRQHFVEYFPAHFYDLRYQNRIPDPCNIGLN   167
sp|P30902|ATP7_YEAST     ETESLVSKELKDLQSTLDNIQSARPFDELTVDDLTKIKPEIDAKVEE------MVKKGKW   159
sp|P13620|ATP5H_BOVIN    ----------QEYEKELEKMRNIIPFDQMTIEDLNEVFPETKLDKKKYPYWPHRPIETL-   161
                                   :: :.  ::  .  *.:.:  :.: :  *    . .

tr|Q17763|Q17763_CAEEL   ETPEIENRFKDYKVLRRADKVDDH    191
sp|P30902|ATP7_YEAST     DVPGYKDRFGNLNVM---------    174
sp|P13620|ATP5H_BOVIN    ------------------------    161
```

Subunit F_6

```
tr|O16517|O16517_CAEEL      -----MFRAVQSV----RS---L-------ST---TAACRQDLIQQTFVTKIREIAK---      35
sp|Q12349|ATP14_YEAST       -----MFPIASRRILLNASVLPLRLCNRNFTTTRISY----NVIQDLYLRELKDTKLAPS      51
sp|P02721|ATP5J_BOVIN       MILQRLFRLSSAV----QSAISV-SWRRNIGITAVAFNKELDPVQKLFVDKIREYRTK--      53
                                 :*   .       *   :            :     : :*. :: ::::

tr|O16517|O16517_CAEEL      ---NAGNLANSDPAVKKALQEELNRLATKFQLANADVVSKLPTNFEAAKVDSAVQSALE      91
sp|Q12349|ATP14_YEAST       TLQDAEGNVKPWNPPQKPNLPELELQ-----GPEALKAYTE--QNVETAHVA---KESEE      101
sp|P02721|ATP5J_BOVIN       --RQTSGGPVDAGPEYQQDLDRELFKLKQMYGKADMNTFPN--FTFEDPKFEVVE-----      104
                                  *.    .*  :  * .   :          ..  :   ..*  :.

tr|O16517|O16517_CAEEL      GQTLASLLEGVKKD-HSEYVASRDAKKAEQAARNAALKQ      129
sp|Q12349|ATP14_YEAST       ---------GESEPIEEDWLVLDDAEETKESH-------      124
sp|P02721|ATP5J_BOVIN       ------------KP-QS----------------------      108
                                        :  ..
```

Subunit OSCP

```
tr|P91283|P91283_CAEEL      MAQ-------LMKRGFSTS--AALAKAQLVKTPIQVHGVEGRYAAALYSAGHKQNKLDQI      51
sp|P05626|ATPF_YEAST        ------MSMSMGVRGLALRSVSKTLFSQGVRCPSMVIGA--RYMSST---PEKQT-----      44
sp|P13621|ATPO_BOVIN        MAALAVSGLSQQVRCFSTS--VVRPFAKLVRPPVQIYGIEGRYATALYSAASKQNKLEQV      58
                                         * ::         :: *: *  : *   ** ::      **.

tr|P91283|P91283_CAEEL      STDLNNVRSVYKDNKKFQEFVLDPTLKANKK----------KTAIEAI-----------      89
sp|P05626|ATPF_YEAST        ------------DPKAKANSIINAIPGNNILTKTGVLGTSAAAVIYAISNELYVINDESI      92
sp|P13621|ATPO_BOVIN        EKELLRVGQILKEPKM-AASLLNPYVKRSVK----------VKSLSDM-----------      95
                                        : *     :::     .               :  :

tr|P91283|P91283_CAEEL      --STKLGLTKETGNFLGLLA---ENGRLNKLESVV-----------SSFESI---MRAH      129
sp|P05626|ATPF_YEAST        LLLTFLGFTGLVAKYLAPAYKDFADARMKKVSDVLNASRNKHVEAVKDRIDSVSQLQNVA      152
sp|P13621|ATPO_BOVIN        --TAKEKFSPLTSNLINLLA---ENGRLTNTPAVI-----------SAFSTM---MSVH      135
                               :   ::  ..: :        :.*:.:   *:            . :.::     .

tr|P91283|P91283_CAEEL      -RGELFVQVTS-AEELSSS-----NQK----ALSDALSKIGKSGQKLT-V-TYAVKPSIL      176
sp|P05626|ATPF_YEAST        ETTKVLFDVSKETVELESEAFELKQKVELAHEAKAVLDSWVRYEASLRQLEQRQLAKSVI      212
sp|P13621|ATPO_BOVIN        -RGEVPCTVTT-ASALDEA-----TLT----ELKTVLKSFLSKGQVLK-L-EVKIDPSIM      182
                               ::   *:. :  *..               . .*..       *  :    :  *::

tr|P91283|P91283_CAEEL      GGLVVTIGD-----KYVDLSIASRVKKYKDALATAI      207
sp|P05626|ATPF_YEAST        SRVQSELGNPKFQEKVLQQSIS-EIEQLLSKLK---      244
sp|P13621|ATPO_BOVIN        GGMIVRIGE-----KYVDMSAKTKIQKLSRAMREIL      213
                            . :   :*:     * :: *   .:::    :
```

Figure S4. Multiple sequence alignment for dimer interface and peripheral stalk subunits. Comparisons were made between *C. elegans*, *S. cerevisiae* (Baker's yeast strain ATCC 204508 / S288c) and *B. taurus* using Clustal Omega at EMBL-EBI (3–5). In all cases the complete protein sequence, including any possible presequences, was used. In the alignment output, an asterisk (*) indicates a perfect alignment, a colon (:) indicates a site belonging to a group exhibiting strong similarity, and full stop (.) indicates a site belonging to a group exhibiting weak similarity. Residues are coloured according to their biophysical properties. Small and hydrophobic residues are coloured red, acidic residues are coloured blue, basic residues are coloured magenta, and hydroxyl, sulfhydryl, amine and glycine residues are coloured green. Extensions in *C. elegans* subunits relative to both the *S. cerevisiae*

and *B. taurus* homologues are underlined in black, deletions are underlined in maroon. Where subunits have multiple isomers, the isomer used in the homology model is used for alignment.

Subunit e (Q21732)

MSAPLKHPNAVVLQPPTVTISPLIRFGRYAALSLGVVYGFFRLRQIREYHADIREWDHEKAVAAAEEA
AKKKKWLAKDEMRYLMQVVNIPFEEGVKQFGVADLYKED

Subunit f (Q22021)

MAWFRPPPPHTQLRPWVPDAIFIPISRAVERVGVFFYNRVLNKTEVGLFDKRWNKNVHGPYCHWRY
YGKLDTKFMDVKLGDLPAWMARREKTPSAFYNEFMRNIWRVHNLYYSGPVYNNTVKVIFRFIFAYSF
LNWLVKSHRYVDFQKTMYHW

Subunit g (isoform 2) (Q18803)

MAAPKLGFFEKIANLTGALYRHQHAQFPRRFAILKAVGKHELAPPRQADWPAIKADWAKVQSFIQTG
GYKNLSIREGLVYTAVTLEVVFWFFVGEMIGRRYIFGYLVPADYVSKSTKKTVKEQEALAALEN

Subunit b (isoform 2) (Q19126)

MSLSRCLPLGQNARVIIIPARLAHAASTQAAAATDDAPNFFQKLAHRFQGVPLKGEAHAPKSMFEDCN
KEWSAPEPLPAIPKDFKEHPDRDLVNYPYPARPMYPPKSRLLMMPDSWFTPFQKVTGVSGPYLFFG
GLFAFLVNKELWVFEEQGHMTVGWILFYLLVTRTAGYKIDQGLYNGYQERVNFFKGLIQEDLKEAVEF
KKTSAKQTESLNSIKESYPTALKESMALQLEATYRKNVQSVATELKRRIDYLKETEESKARVEREQLLK
LINSEVDKEFSDRSFKDKYLQNAIQQLKGLNVQL

Subunit d (Q17763)

MSGAAKRVATSSVNWSKLAERLVPEHAAELTRVKGVSGTFQSAVSQLPADLPKIDFAALKKALPAHS
AVLDSLQKQYESVKIPYGEVPAEYLKEVDQWVDYNNARIKLHEVKVADGLQEAKKVEEKWAKAPPVE
HFDRQHFVEYFPAHFYDLRYQNRIPDPCNIGLNETPEIENRFKDYKVLRRADKVDDH

Subunit F_6 (O16517)

MFRAVQSVRSLSTTAACRQDLIQQTFVTKIREIAKNAGNLANSDPAVKKALQEELNRLATKFQLANAD

VVSKLPTNFEAAKVDSAVQSALEGQTLASLLEGVKKDHSEYVASRDAKKAEQAARNAALKQ

Figure S5. Mass spectrometry data for *C. elegans* ATP synthase subunits with significant extensions. The sequence for each subunit of interest is shown and identified with a Uniprot code. The mitochondrial targeting sequences as predicted by MitoFates (6) and TargetP-2.0 (7) are coloured red. The *C. elegans* specific extensions (revealed in sequence alignments from Fig. S4) are highlighted in yellow. Peptides identified by mass spectrometry are underlined. Where subunits have multiple isomers, the isomer used in the homology model is shown.

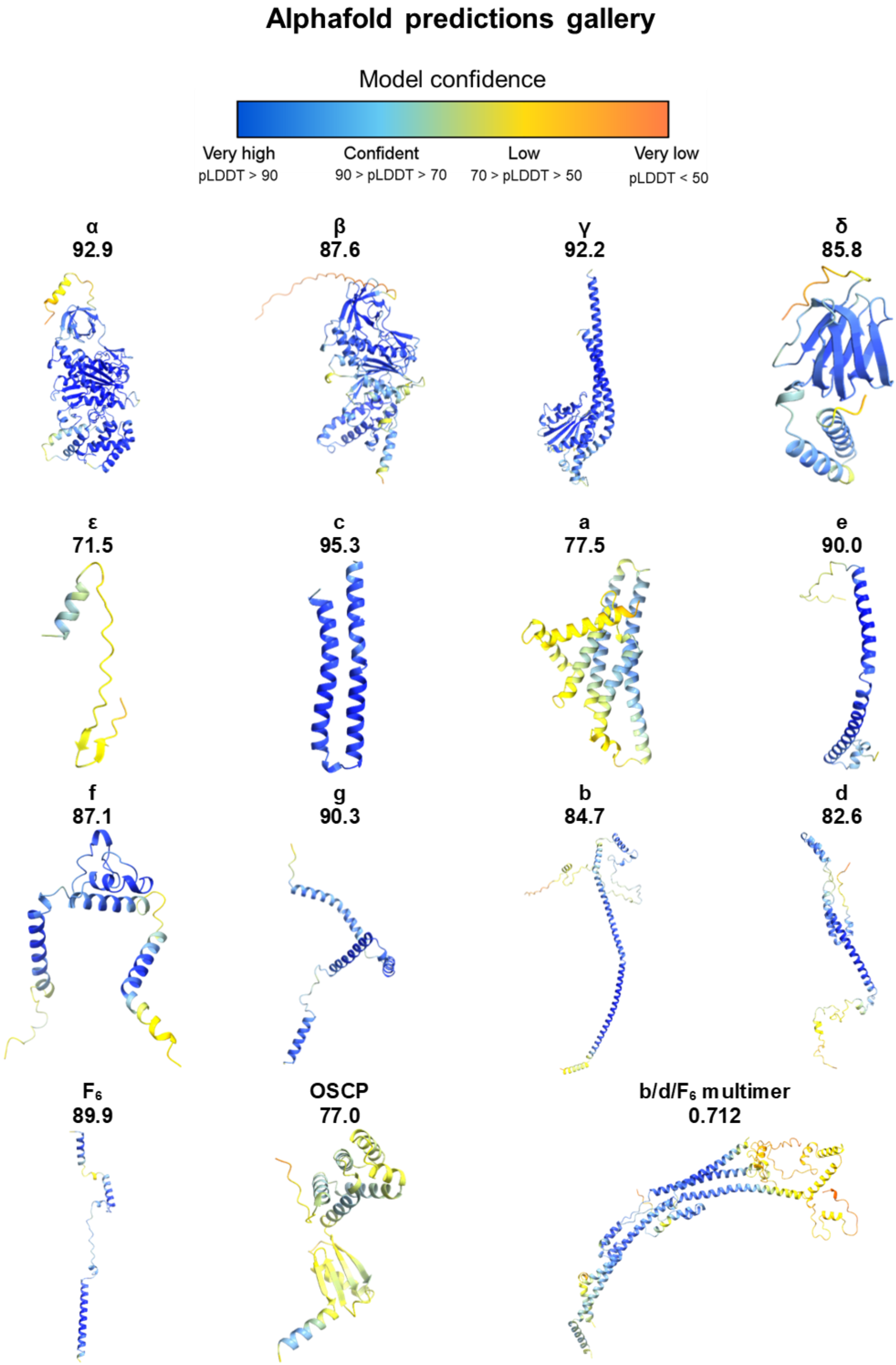

Figure S6. AlphaFold predictions gallery

AlphaFold predictions (8) for each *C. elegans* ATP synthase subunit, coloured by pLDDT score per

residue. Mature sequences were submitted to AlphaFold Any presequences predicted by MitoFates (6) and Predict-P2.0 (7) were removed before submission of mature sequences to AlphaFold. The pLDDT score is a per-residue measure of local confidence on a scale from 0 – 100. The structure of subunits b d and F_6 were predicted as a multimer using AlphaFold multimer (9). The confidence measure for predictions (9) is similar but modified to score interactions between residues of different chains. It is calculated using a weighted combination of predicted-TM score (pTM) and interface predicted-TM score (ipTM) and has a scale from 0-1. The appropriate mean confidence score for each AlphaFold / multimer prediction is shown beneath each subunit name.

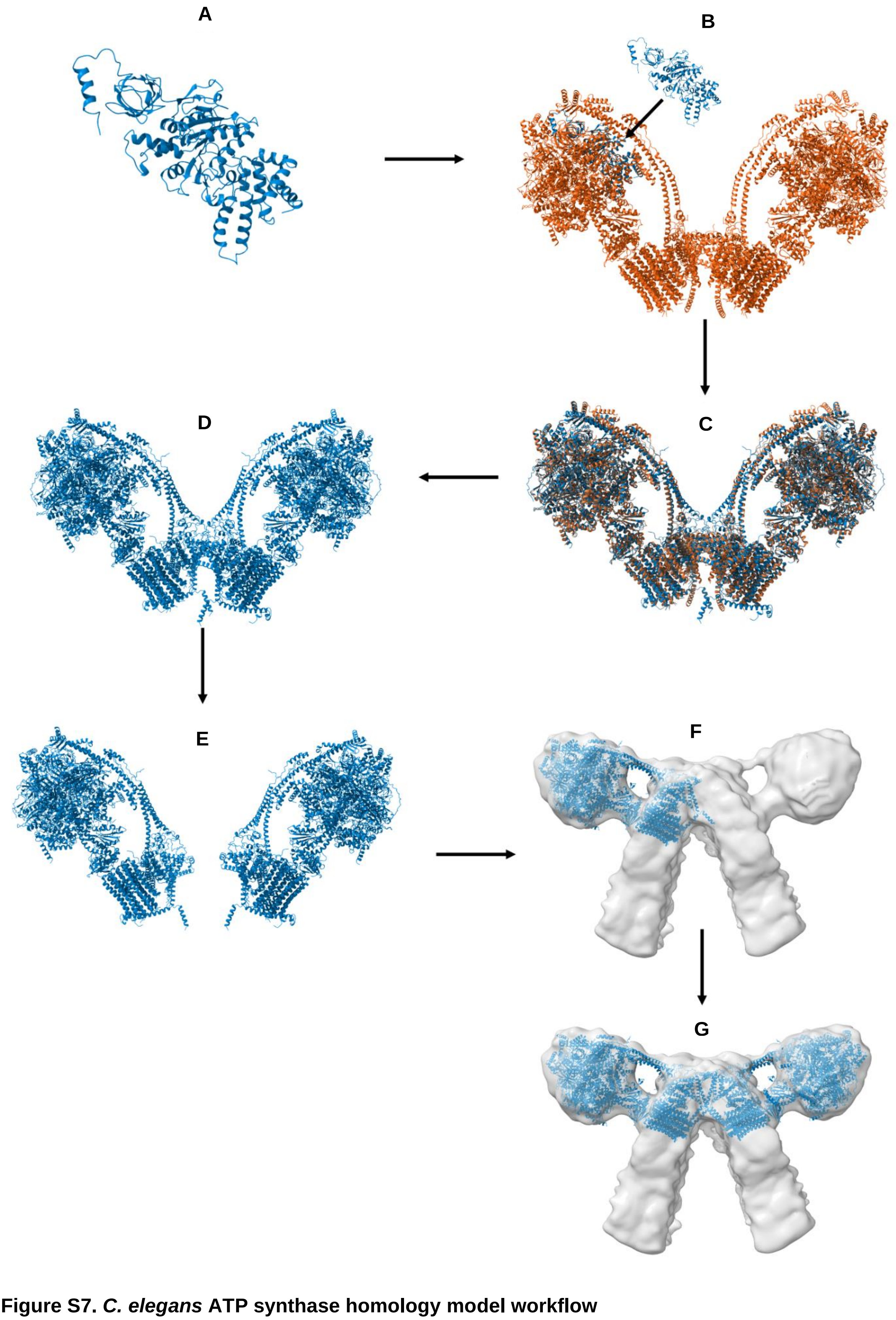

Figure S7. *C. elegans* ATP synthase homology model workflow

(A) AlphaFold was used to predict structures of all mature *C. elegans* ATP synthase subunits; the

example shown is the prediction for subunit α. **(B)** Predicted models were sequentially fitted into the *B. taurus* ATP synthase model [PDB 7AJB] (10) used as a scaffold using MatchMaker in ChimeraX (11). **(C)** The resulting homology model (blue) after all subunits have been fitted to the scaffold provided by 7AJB (orange). **(D)** The homology model without the *B. taurus* scaffolding. **(E)** The *C. elegans* ATP synthase dimer was split into separate monomers. **(F)** The monomers were fitted sequentially into the sub-tomogram average of the *C. elegans* ATP synthase using matchmaker in ChimeraX (11) to obtain the correct dimer angle. **(G)** The final homology model of the *C. elegans* ATP synthase dimer fitted into the sub-tomogram average.

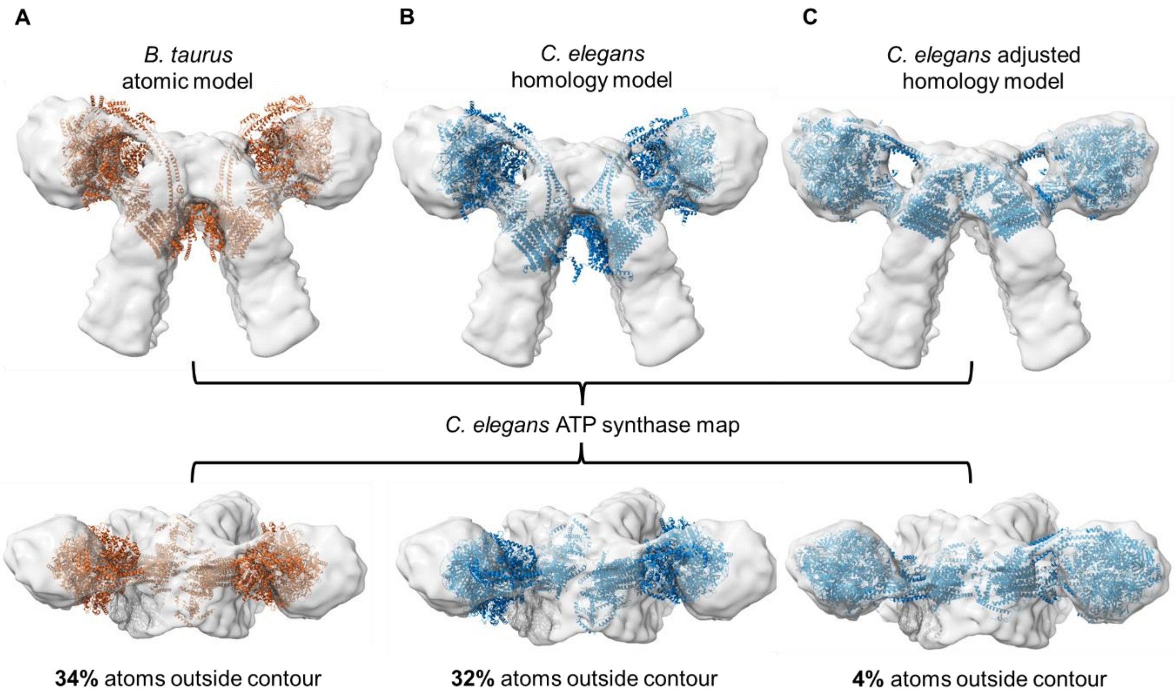

Figure S8. Comparison of different models fitted to the *C. elegans* ATP synthase dimer map. Different ATP synthase dimer models were fitted into the *C. elegans* ATP synthase *in situ* map. All models were fitted into the map at threshold 0.0429 in ChimeraX (11), and the percentage of atoms outside the contour is shown for each model. **(A)** The purified *B. taurus* ATP synthase dimer atomic model [PDB 7AJB] (10) used as a scaffold shows a poor fit, with 34% of atoms outside the contour. **(B)** The *C. elegans* ATP synthase dimer homology model following scaffolding to the *B. taurus* model also shows a poor fit, with 32% of atoms outside the contour. **(C)** Sequential fitting of monomers from the *C. elegans* homology model shows an improved fit, with only 4% of atoms outside the contour.

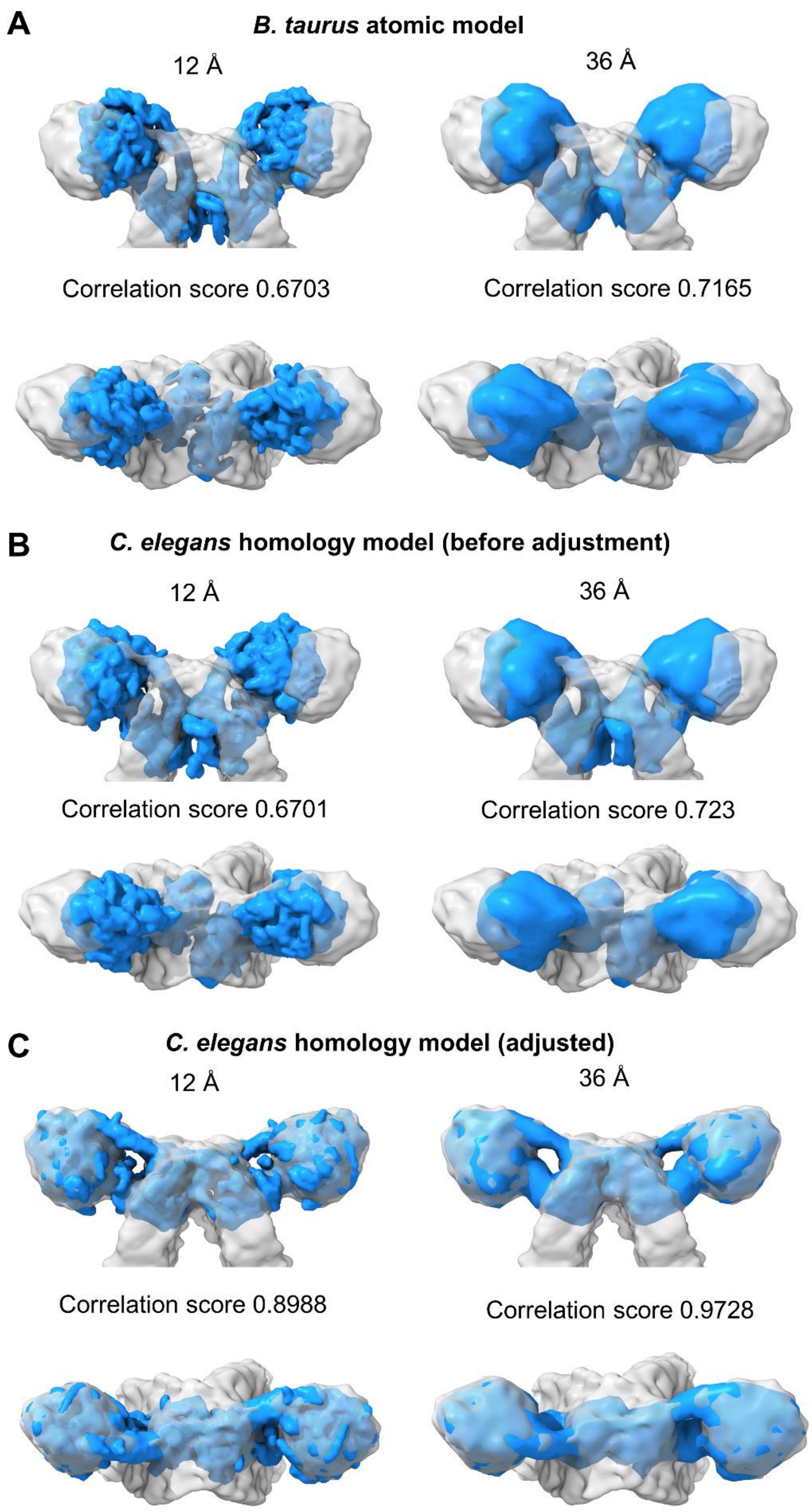

Figure S9. The *C. elegans* homology model fitted to the *C. elegans* ATP synthase dimer sub-tomogram averaging map. Using the molmap command in Chimera X (11), the PDB of the *C. elegans*

homology model was converted into an MRC map at both 12 Å and 36 Å resolution. Converted molmap maps (blue) were then fitted to the sub-tomogram averaging map of the *C. elegans* dimer (grey) at equivalent threshold levels. Correlation scores between the homology model and sub-tomogram averaging maps are displayed. **(A)** Maps of the 7AJB *B. taurus* ATP synthase atomic model (10) used as a scaffold fitted to the sub-tomogram average for reference**.** **(B)** Maps of the *C. elegans* original homology model (without adjusting for dimer angle) fitted to the sub-tomogram average. **(C)** Maps of the dimer angle adjusted *C. elegans* homology model fitted to the sub-tomogram average**.**

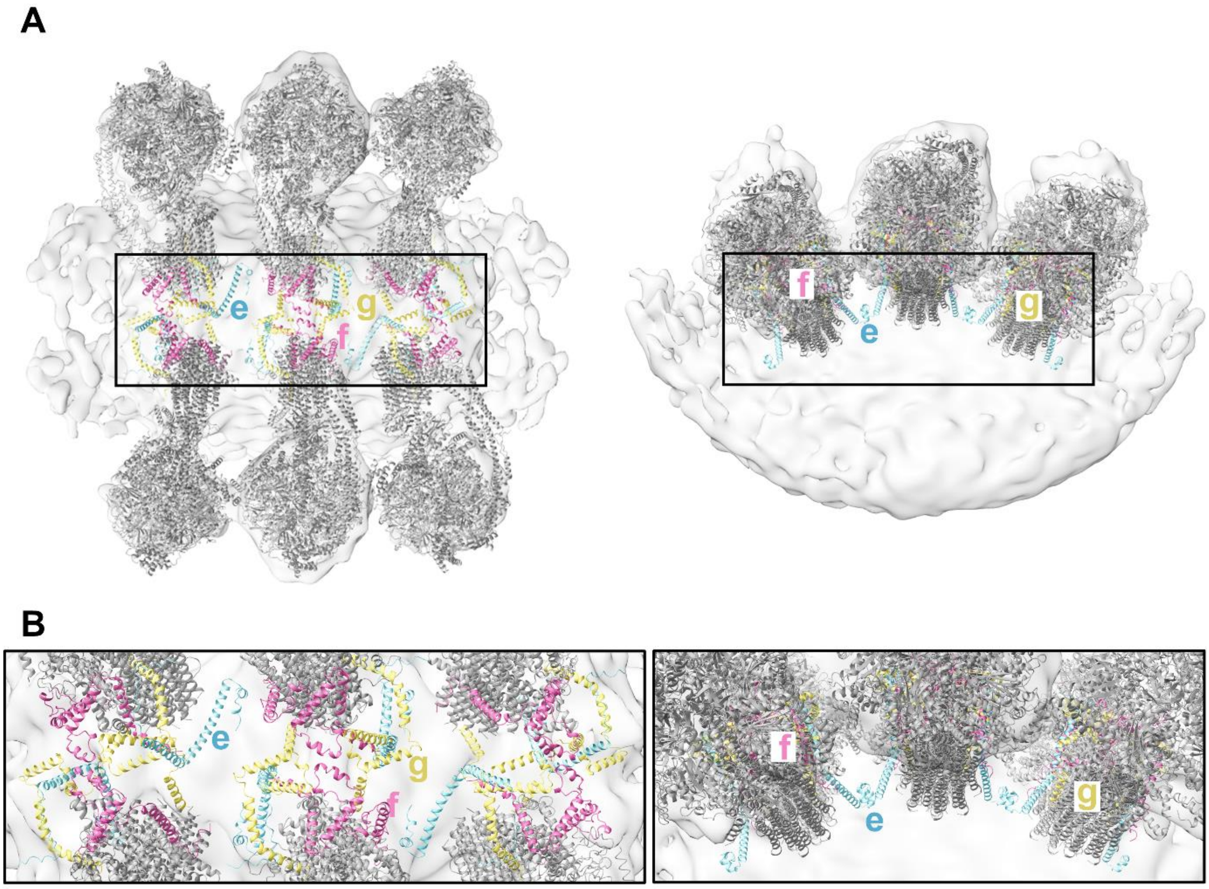

Figure S10. Inter-dimer interactions mediated by subunits e and g in *C. elegans* ATP synthase dimer rows. (A) Top-down view (left) and side view (right) of the *C. elegans* ATP synthase homology model (grey) fitted to each dimer pair in the unmasked sub-tomogram average of the *C. elegans* dimer. Dimer interface subunits are colored (e, pale blue; f, pink; g, yellow) to highlight inter-dimer interactions mediated by subunits e and g. **(B)** Close up view of inter-dimer interactions boxed in panel A.

C. elegans	*S. cerevisiae*	*B. taurus*
**F_1 head**		
α	α/Atp1	α
β	β/Atp2	β
**F_o head**		
γ	γ/Atp3	γ
δ	δ/Atp16	δ
ε	ε/Atp15	ε
**Peripheral stalk**		
b	b/Atp4	b
d	d/Atp7	d
F_6	h/Atp14	F_6
OSCP	OSCP/Atp5	OSCP
**F_o motor**		
a	a/Atp6	a
c	c/Atp9	c
**Type I dimer-specific subunits**		
e	e/Atp21	e
f	f/Atp17	f
g	g/Atp20	g
-	i/j/Atp18	6.8PL/ j
-	k/Atp19	DAPIT/ k
-	8/Atp8	A6L/ATP8

Table S1. Nomenclature for homologues of ATP synthase subunits

Nomenclature for yeast and mammalian species are described as detailed by Song and Pfanner (12). In this work, we use primarily the mammalian nomenclature, which is also the standard used to describe *C. elegans* subunits. Exceptions to this are in our comparisons between *C. elegans* and *S. cerevisiae* dimers, where we use the *S. cerevisiae* naming system to describe subunits missing in worms.

Subunit name	*C. elegans* Uniprot Accession Number	Mean pLDDT (or weighted pTM & ipTM)[1]	RMSD between pruned atom pairs[2]	RMSD across all atom pairs	Sequence alignment score[3]
α	Q9XXK1	92.9066444	0.586	1.009	2171.8
β	P46561	87.5983733	1.161	3.202	2050.1
γ	Q95XJ0	92.17635904	0.743	1.764	978.4
δ	Q09544	85.82596715	0.901	0.941	368.7
ε[4]	O16298	65.4116052			
	P34539	71.51756013	0.631	6.924	39
c	Q9BKS0	95.27668763	0.558	0.62	332.8
e	Q21732	89.96603434	0.609	10.661	143.9
f	Q22021	87.12810426	1.231	6.257	167.2
g	Q18803	90.2590889	1.346	2.598	207.2
a	P24888	77.54888203	1.073	4.582	322.9
b	Q20053	84.43326886			
	Q19126	84.74422485	1.076	8.068	441.4
d	Q17763	82.60642993	1.175	4.902	296.7
F_6	O16517	89.9038886	0.79	6.717	76.2
OSCP	P91283	76.95722866	1.09	1.557	517.7
	Q7JNG1	76.43462181			
b,d,F_6 multimer	Q19126, Q17763, O16517	0.712090029	1.076	8.068	441.4

Table S2. Metrics to assess confidence and fit of AlphaFold predicted structures

[1] pLDDT scores are shown for subunits where structure was predicted individually, a weighted pTM and iPTM score is shown for a complex of subunits predicted using AlphaFold multimer. The pLDDT score is a per-residue measure of local confidence on a scale from 0 – 100. The predicted-TM score (pTM) and interface predicted-TM score (ipTM) and has a scale from 0-1.

[2] RMSD (Root Mean Square Deviation) is a measure of the similarity between two superimposed atomic coordinates, in this case for the predicted *C. elegans* subunits and the model of the *B. taurus* ATP synthase dimer.

[3] Sequence alignment score between *C. elegans* and *B. taurus.*

[4] Where a subunit has more than one isoform, the version with the highest pLDDT score was used to build the homology model. RMSD and sequence alignment scores are only shown for the selected protein. In the case of subunit b, the isoform with the highest pLDDT score is also the only isoform expressed in somatic tissues (13).

	B. taurus atomic model [PDB 7AJB (10)]	Original[5] *C. elegans* homology model	Adjusted[6] *C. elegans* homology model
PDB % atoms outside contour[7]	34	32	4
MRC map[8] correlation score	0.7165	0.723	0.9728

Table S3. Metrics to assess fit of atomic detail models to *C. elegans* ATP synthase dimer sub-tomogram averaging map.

[5] Homology model following scaffolding of AlphaFold predicted *C. elegans* subunits onto the *B. taurus* atomic model without adjusting for dimer angle.
[6] Homology model following fitting of dimer angle adjusted *C. elegans* ATP synthase monomers to the *C. elegans* ATP synthase sub-tomogram averaging map.
[7] This value is given by Chimera when fitting a PDB model to a map using the “fit in map” command.
[8] MRC map generated from PDB’s using molmap command in ChimeraX (11). This metric shows level of correlation between molmap map and our sub-tomogram average at the same resolution (36 Å).

Supplementary Movie 1 (separate file). Movie showing a 360° rotation about the y-axis of a single segmented *C. elegans* mitochondrion from the upper panel of Fig. 3A. An image sequence of 100 PNG files was collected in IMOD, and the sequence montaged into a10fps AVI file in Image J (14).

Supplementary Movie 2 (separate file). Movie showing a 360° rotation about the y-axis of a single segmented *S. cerevisiae* mitochondrion from the lower panel of Fig. 3A. An image sequence of 100 PNG files was collected in IMOD, and the sequence montaged into a10fps AVI file in Image J (14).

References

1. Spikes, TE. (2017) Structural studies of the mitochondrial F-ATPase. PhD thesis. University of Cambridge. 10.17863/CAM.21471

2. Runswick MJ, Bason J V., Montgomery MG, Robinson GC, Fearnley IM, Walker JE. (2012) The affinity purification and characterization of ATP synthase complexes from mitochondria. *R. Soc. Open Biol.* **3** doi: 10.1098/rsob.120160

3. Sievers F, Wilm A, Dineen D, Gibson TJ, Karplus K, Li W, et al. (2011) Fast, scalable generation of high-quality protein multiple sequence alignments using Clustal Omega. Mol. Syst. Biol. **7**, 539 10.1038/msb.2011.754.

4. Goujon M, McWilliam H, Li W, Valentin F, Squizzato S, Paern J, *et al*. (2010) A new bioinformatics analysis tools framework at EMBL-EBI. *Nucleic Acids Res.* **38**, W695–9 10.1093/nar/gkq313

5. McWilliam H, Li W, Uludag M, Squizzato S, Park YM, Buso N, *et al*. (2013) Analysis Tool Web Services from the EMBL-EBI. *Nucleic Acids Res.* **41**, W597–600 10.1093/nar/gkt376

6. Fukasawa Y, Tsuji J, Fu SC, Tomii K, Horton P, Imai K. (2015) MitoFates: Improved Prediction of Mitochondrial Targeting Sequences and Their Cleavage Sites. *Mol. Cell. Proteomics.* **14**, 1113–26 10.1074/mcp.M114.043083

7. Armenteros JJA, Salvatore M, Emanuelsson O, Winther O, Von Heijne G, Elofsson A, et al. (2019) Detecting sequence signals in targeting peptides using deep learning. *Life Sci. Alliance*. **2**, e201900429 10.26508/lsa.201900429

8. Jumper J, Evans R, Pritzel A, Green T, Figurnov M, Ronneberger O, *et al*. (2021) Highly accurate protein structure prediction with AlphaFold. *Nature.* **596**, 583–9 10.1038/s41586-021-03819-2

9. Evans R, O'neill M, Pritzel A, Antropova N, Senior A, Green T, et al. (2021) Protein complex prediction with AlphaFold-Multimer. *bioRxiv.* 10.1101/2021.10.04.463034. Posted March 10, 2022.

10. Spikes TE, Montgomery MG, Walker JE. (2021) Interface mobility between monomers in dimeric bovine ATP synthase participates in the ultrastructure of inner mitochondrial membranes. *Proc. Natl. Acad. Sci. USA.* **118**, e2021012118 10.1073/pnas.2021012118

11. Pettersen EF, Goddard TD, Huang CC, Meng EC, Couch GS, Croll TI, *et al*. (2021) UCSF ChimeraX: Structure visualization for researchers, educators, and developers. *Protein Sci*. **30**, 70–82 10.1002/pro.3943

12. Song J, Pfanner N, Becker T. (2018) Assembling the mitochondrial ATP synthase. *Proc. Natl. Acad. Sci. USA.* **115**, 2850–2 10.1073/pnas.1801697115

13. Kawasaki I, Hanazawa M, Gengyo-Ando K, Mitani S, Maruyama I, Iino Y. (2007) ASB-1, a germline-specific isoform of mitochondrial ATP synthase b subunit, is required to maintain the rate of germline development in *Caenorhabditis elegans*. *Mech. Dev.* **124**, 237–51 10.1016/j.mod.2006.11.004

14. Schneider CA, Rasband WS, Eliceiri KW. (2012) NIH Image to ImageJ: 25 years of image analysis. *Nat. Methods.* **9**, 671–5 10.1038/nmeth.2089
